# Plant-derived virus-like particle vaccines drive cross-presentation of influenza A hemagglutinin peptides by human monocyte-derived macrophages

**DOI:** 10.1038/s41541-019-0111-y

**Published:** 2019-05-15

**Authors:** Alexander I. Makarkov, Makan Golizeh, Elizabeth Ruiz-Lancheros, Angelica A. Gopal, Ian N. Costas-Cancelas, Sabrina Chierzi, Stephane Pillet, Nathalie Charland, Nathalie Landry, Isabelle Rouiller, Paul W. Wiseman, Momar Ndao, Brian J. Ward

**Affiliations:** 1 Division of Experimental Medicine, Department of Medicine, McGill University, 1001 Décarie Street, Montréal, QC H4A 3J1 Canada; 2 Infectious Diseases and Immunity in Global Health Program, Research Institute of McGill University Health Centre, Glen Site, 1001 Décarie Street, Montréal, QC H4A 3J1 Canada; 3 Department of Chemistry, McGill University, 801 Sherbrooke St. W, Montreal, QC H3A 0B8 Canada; 4 Department of Physiology, McGill University, 3655 Promenade Sir William Osler, Montreal, QC H3G 1Y6 Canada; 5 Department of Anatomy & Cell Biology, Faculty of Medicine, Groupe de Recherche Axé sur la Structure des Protéines (GRASP), Groupe d’Étude des Protéines Membranaires (GEPROM), McGill University, 3640 University Street, Montreal, QC H3A 2B2 Canada; 6 Research Institute of McGill University Health Centre, Montreal General Hospital, 1650 Cedar Av, Montréal, QC H3G 1A4 Canada; 7 Medicago Inc., 1020 Route de l’Église, Bureau 600, Québec, QC G1V 3V9 Canada; 8 Department of Biochemistry and Molecular Biology and Bio21 Molecular Science and Biotechnology Institute, The University of Melbourne, Parkville, VIC 3010 Australia; 9 Department of Physics, McGill University, 3600 University St., Montreal, QC H3A 2T8 Canada; 10 Department of Medicine, Division of Infectious Diseases, Faculty of Medicine, McGill University, Montreal, QC H4A 3J1 Canada; 11 Present Address: Medicago Inc., 1020 Route de l’Église, Bureau 600, Québec, QC G1V 3V9 Canada

**Keywords:** Recombinant vaccine, Cellular immunity, Influenza virus, Vaccines

## Abstract

A growing body of evidence supports the importance of T cell responses to protect against severe influenza, promote viral clearance, and ensure long-term immunity. Plant-derived virus-like particle (VLP) vaccines bearing influenza hemagglutinin (HA) have been shown to elicit strong humoral and CD4^+^ T cell responses in both pre-clinical and clinical studies. To better understand the immunogenicity of these vaccines, we tracked the intracellular fate of a model HA (A/California/07/2009 H1N1) in human monocyte-derived macrophages (MDMs) following delivery either as VLPs (H1-VLP) or in soluble form. Compared to exposure to soluble HA, pulsing with VLPs resulted in ~3-fold greater intracellular accumulation of HA at 15 min that was driven by clathrin-mediated and clathrin-independent endocytosis as well as macropinocytosis/phagocytosis. At 45 min, soluble HA had largely disappeared suggesting its handling primarily by high-degradative endosomal pathways. Although the overall fluorescence intensity/cell had declined 25% at 45 min after H1-VLP exposure, the endosomal distribution pattern and degree of aggregation suggested that HA delivered by VLP had entered both high-degradative late and low-degradative static early and/or recycling endosomal pathways. At 45 min in the cells pulsed with VLPs, HA was strongly co-localized with Rab5, Rab7, Rab11, MHC II, and MHC I. High-resolution tandem mass spectrometry identified 115 HA-derived peptides associated with MHC I in the H1-VLP-treated MDMs. These data suggest that HA delivery to antigen-presenting cells on plant-derived VLPs facilitates antigen uptake, endosomal processing, and cross-presentation. These observations may help to explain the broad and cross-reactive immune responses generated by these vaccines.

## Antigen processing: Plant-derived virus-like particles

Producing vaccines in plants can have several important advantages, including scalability and relatively low cost. Brian J. Ward and colleagues at McGill University examine the intracellular processing of a plant-derived virus-like particle (VLP) expressing influenza hemagglutinin H1 (H1-VLP) and compare this systematically with soluble monomeric H1. Human monocyte-derived macrophages rapidly take up soluble H1 via degradative pathways resulting in its poor presentation by MHC class I. In contrast, multiple endocytic and pinocytic mechanisms are used to internalize H1-VLP, including handling by non-degradative pathways which favors efficient cross-presentation by MHC class I. This specialized intracellular handling of plant-derived VLPs might underlie their ability to stimulate robust CD8^+^ T cell responses.

## Introduction

The cellular arm of the adaptive immune response is increasingly recognized as important for both recovery and long-term protection from influenza viruses. CD4^+^ T cells provide support for antibody production and maturation as well as the induction of cytotoxic CD8^+^ T cells (CTL) that target infected cells for elimination.^1^ Although limited in scope, a recent human challenge study with both H1N1 and H3N2 viruses proposed pre-existing poly-functional CD4^+^ T cells as a novel correlate of protection.^2^ Although influenza-specific CTLs cannot prevent disease, they can reduce both the severity and duration of infection.^3^ T cell responses may be particularly important for vulnerable populations such as young children and the elderly.^4,5^ The most commonly used influenza vaccines based on detergent-split virions typically elicit a strong antibody response but are weak inducers of cellular immunity.^6^ Although live attenuated vaccines elicit T cell responses, systemic humoral responses are often weak, and interference from pre-existing immunity makes these vaccines less effective after early childhood.^7^ A vaccine that elicits both strong antibody and cell-mediated responses might bring us closer to the control of influenza in the human population.

Plant-derived virus-like particle (VLP) vaccines are produced by *Agrobacterium*-mediated transient expression of influenza hemagglutinin (HA) proteins in *Nicotiana benthamiana*. Influenza HA-bearing VLPs self-assemble and do not require accessory proteins for budding from the plant cells.^8^ The plant-derived HA-VLPs are ~100 nm in size.^8^ Each particle has 30–50 homotrimer HA “spikes” inserted into a lipid bilayer envelope of plant cell origin.^9,10^ The HA monomers on the plant-derived VLPs are ~72 kDa corresponding to the uncleaved HA0 form.^8^ Plant-derived H1 proteins contain six *N*-glycosylation sites, located either in the HA1 globular head or the HA2 stem region that carry complex or hybrid glycans containing core α(1,3)-fucose or β(1,2)-xylose epitopes.^11^ Traces of plant proteins can be detected by mass spectrometry (MS), many of which were previously identified in *Nicotiana tabacum* lipid rafts, suggesting that the mechanism of VLP formation in plants is similar to the natural process of influenza virus assembly in the mammalian host cells.^9,11^ The plant-based transient expression system allows rapid and large-scale production of influenza HA-based vaccine at relatively low cost, addressing several of the challenges for vaccine production in a pandemic (i.e., speed and scalability) and representing an alternative to the currently available manufacturing platforms for seasonal vaccines.^8^ Plant-derived VLPs recapitulate the key features of native influenza virions such as sialic acid-mediated adherence and internalization by target cells, fusion of the VLP envelope with endosomal membranes, and rapid induction of an innate immune response.^10,12,13^ These vaccines have been shown to elicit strong and cross-reactive antibody responses against both seasonal and pandemic influenza strains in animal models and human trials.^14–16^ They also induce polyfunctional and cross-reactive HA-specific CD4^+^ T cell responses.^14,15,17^ Simultaneous administration of a plant-derived H1-VLP vaccine with ovalbumin (OVA) was recently shown to elicit an OVA-specific CD8^+^ T cell response in C57BL/6 mice.^18^ The subcellular mechanisms that account for the unusual immunogenicity of the plant-derived VLP-based vaccines are not yet well understood.

In the current work, we demonstrated that human monocyte-derived macrophages (MDMs) internalize H1-VLPs using both clathrin-mediated and clathrin-independent endocytosis (CME and CIE respectively) as well as macropinocytosis and, probably, phagocytosis. Soluble H1 was internalized almost exclusively by CME and was trafficked predominantly to the high-degradative late endosome/endolysosome compartment. In contrast, a substantial portion of H1 delivered by VLP was retained in low-degradative static early and/or recycling endosomes where the HA co-localized with major histocompatibility complex (MHC) I protein. Immunoprecipitation of MHC I and high-resolution MS revealed a large number of HA-derived peptides in MDMs exposed to H1-VLP but not soluble H1. These findings demonstrate that intracellular processing of influenza HA by human MDMs is very different when the protein is delivered by VLP or in a soluble form. These observations help to explain the dual humoral and CD4^+^ responses seen in humans with the plant-derived VLP vaccines and raise the possibility that cross-presentation of HA peptides to CD8^+^ T cells may also occur.

## Results

### Characterization of influenza HA presented on H1-VLPs and recombinant soluble H1 protein

**Fig. 1:**
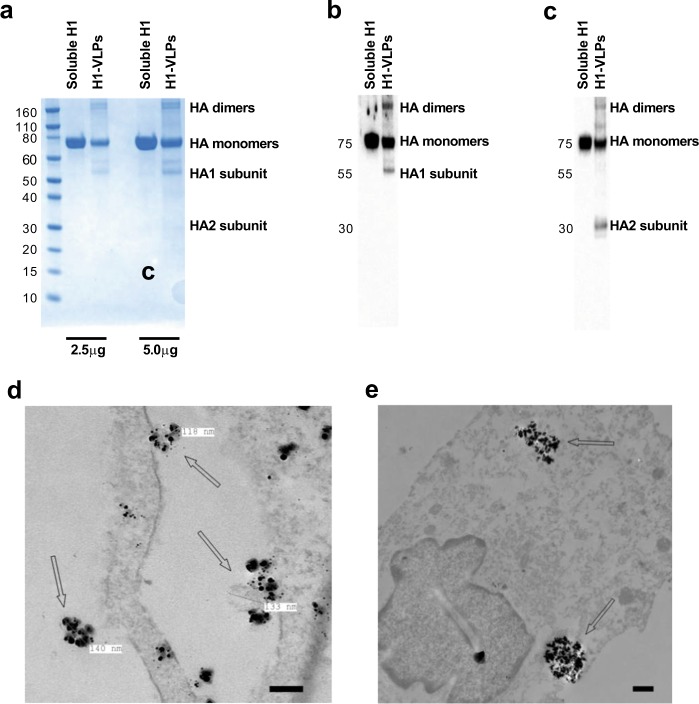
Characterization of influenza HA presented on H1-VLPs and recombinant soluble H1 protein. **a** SDS-PAGE of H1-VLPs and soluble H1 protein (2.5 and 5.0 µg per sample) followed by Coomassie blue staining. Representative image from 3 experiments shown. **b** Immunoblot analysis of H1-VLPs and soluble H1 protein using rabbit polyclonal anti-H1 antibody. Representative image from 3 experiments shown. **c** Immunoblot analysis of H1-VLPs and soluble H1 protein using mouse monoclonal anti-H1 antibody. Representative image from 3 experiments shown. **d** Representative TEM image shows H1-VLPs with nanogold immunolabeled HA. Arrows indicate HA-bearing VLPs surrounded by nanogold particles on the surface of MDM exposed to H1-VLPs. Scale bar–100 nm. **e** Representative TEM image shows endosomal accumulation of nanogold immunolabeled HA in MDM. Arrows indicate HA-loaded endosomes in MDM exposed to H1-VLPs for 15 min. Scale bar–500 nm

To study the uptake and endosomal handling of influenza HA presented on plant-derived H1-VLPs, initial experiments were performed with a commercially available recombinant protein produced in mammalian 293 cells. This comparator consisted of the extracellular and intracellular domains of the H1 of the A/California/07/2009 H1N1 virus but lacked the transmembrane portion, which permitted secretion of the soluble protein from host cells and prevented formation of the HA multimeric structures.^19^ SDS-PAGE followed by Coomassie blue staining (Fig. 1a and Supplementary Fig. 1a) and immunoblot analysis with anti-H1 polyclonal (Fig. 1b and Supplementary Fig. 1b) or monoclonal (Fig. 1c and Supplementary Fig. 1c) antibodies showed that influenza H1 was the predominant protein in both H1-VLP and soluble H1 stocks. HA monomers ~75 kDa (mostly) and HA dimers and trimers, as well as HA1 (~55 kDa) and HA2 (~30 kDa) subunits, were observed in H1-VLP samples. The soluble H1 was present as a single protein ~75 kDa corresponding to uncleaved HA monomers, as expected. No HA degradation products and no non-specific (non-HA) proteins were found in either the H1-VLP or soluble H1 samples. The monoclonal anti-H1 antibody that we used in imaging experiments (both confocal and transmission electron microscopy—TEM) bound equally well to HA in both H1-VLPs and the soluble H1 comparator (Fig. 1c and Supplementary Fig. 1c). The antibody binding epitope was located on the HA2 subunit, as revealed by visualization of uncleaved HA and ~30 kDa (but not ~55 kDa) bands on the H1-VLP immunoblot. The protein concentration of both products measured by BCA assay was consistent with the HA content reported by the respective manufacturers. Negative stain TEM of H1-VLPs showed pleomorphic particles with an average diameter of ~100 nm and spikes on their surface (Supplementary Fig. 1d). VLP-like objects were observed on the surface of MDMs at 5 min after exposure to H1-VLPs but not the soluble H1 (Supplementary Fig. 1e). Nanogold immunostaining with anti-H1 antibody confirmed that these objects were indeed the HA-bearing VLPs. Nanogold-labeled HA was found on the surface of VLP-treated MDMs (Fig. 1d) and inside the endosomes (Fig. 1e).

In summary, plant-derived VLPs bearing influenza HA are pleomorphic particulate structures, similar in size to native influenza virions. The HA molecules on the H1-VLPs form supramolecular complexes, whereas the comparator (soluble H1 protein) is monomeric. Both the H1-VLP and soluble H1 preparations contained primarily HA protein without any unexpected degradation products or non-HA contaminants. The anti-H1 monoclonal antibody used in these studies bound equally well to the HA from both sources.

### H1-VLPs are efficiently internalized by human MDMs

**Fig. 2:**
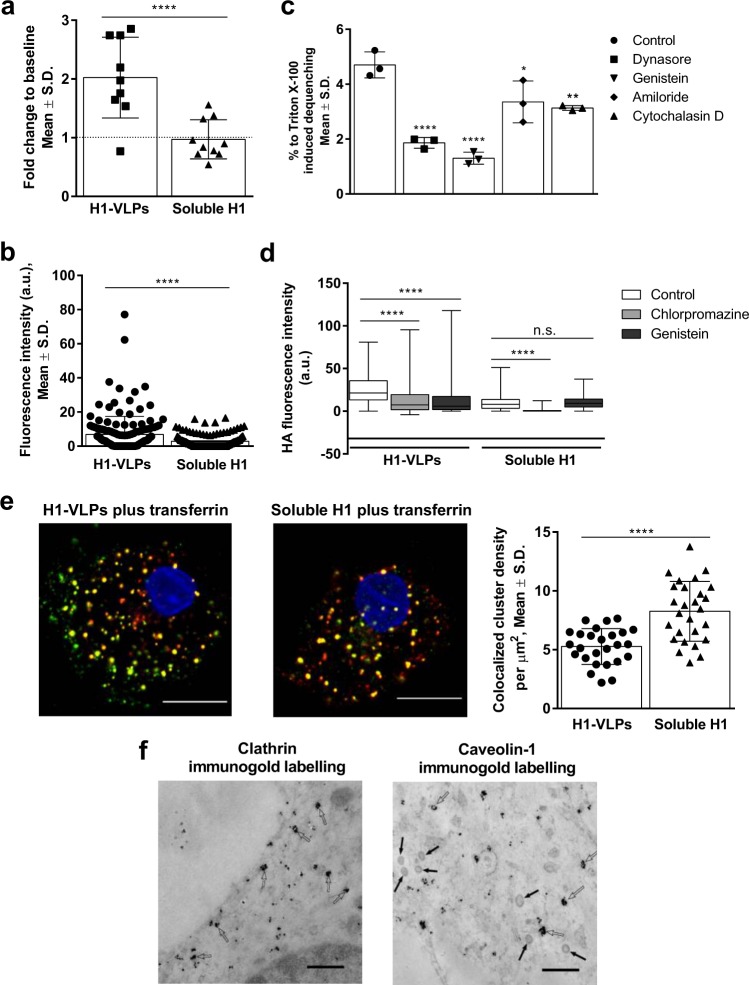
Mechanisms of H1-VLPs and soluble H1 internalization by human MDMs. **a** Number of endocytic vesicles in MDMs exposed to H1-VLPs or soluble H1 for 5 min, normalized against the baseline count (taken as 1, dotted line). Data from two experiments were analyzed. **b** HA internalization by MDMs exposed to either H1-VLPs or soluble H1 for 15 min. The amount of internalized protein was evaluated by the intensity of HA immunofluorescence per cell area on confocal microscopy images. Based on 4 experiments. **c** Effect of endocytosis inhibitors on DiD dequenching by MDMs loaded with DiD-labeled H1-VLPs (at 2 h). Data from 3 experiments were analyzed. **d** Effect of chlorpromazine and genistein on H1-VLPs or soluble H1 internalization by MDMs upon 15 min of exposure. The amount of internalized protein evaluated by the intensity of HA immunofluorescence per cell area on confocal microscopy images. Based on 6 experiments. **e** Colocalization of HA and transferrin in MDMs exposed to H1-VLPs and transferrin (left) or soluble H1 and transferrin (center), and segmentation ICCS colocalization (number of colocalized particles per µm^2^—right). Representative images from 3 experiments shown. Scale bar–10 µm. Green: fluorescently labeled HA, red: transferrin conjugated with CF568 fluorophore (yellow shows colocalization of two proteins), blue: nuclei stained with DAPI. **f** Representative TEM image with nanogold immunolabeled clathrin (left). Open arrows indicate clathrin-coated endocytic vesicles. Representative TEM image with nanogold immunolabeled caveolin-1 (right). Open arrows indicate caveolin-coated endocytic vesicles. Solid arrows indicate unlabeled clathrin-coated endocytic vesicles with typical clathrin spikes. Scale bar–500 nm. Bar graphs present mean ± standard deviation (S.D.); box and whisker plot presents the minimum, maximum, median, and 25th and 75th percentiles. *p < 0.05, **p < 0.01, ****p < 0.0001. n.s.: nonsignificant (**a**, **c**, **d**—one-way ANOVA followed by Tukey’s multiple comparisons post-test; **b**, **e**—Mann–Whitney test)

Classical TEM was used to document early endocytotic events such as formation of endocytic vesicles that are too small to be well-visualized with confocal microscopy.^20^ Exposure of MDMs to H1-VLPs led to rapid activation of the endocytosis machinery. The number of endocytic vesicles doubled during the first 5 min of exposure to H1-VLPs while soluble H1 had no significant effect (Fig. 2a). Intracellular HA immunofluorescence was apparent at 5 min of exposure to H1-VLPs and reached a plateau at 10 min. Further incubation did not change the fluorescence signal (Supplementary Fig. 2a). To eliminate continuous internalization, MDMs were pulsed with either H1-VLPs or soluble H1 for 15 min followed by a 30 min incubation. At 15 min, the fluorescence was 3-fold higher in H1-VLP-treated MDMs compared to soluble H1 (Fig. 2b). Of note, the HA fluorescence intensity in MDMs exposed to H1-VLPs was ~4–6-fold greater than in those exposed to the licensed monovalent split virion H1N1 vaccine (Supplementary Fig. 2b), suggesting that recombinant soluble HA and HA from the split virion vaccine display similar MDM internalization characteristics, and that soluble HA can serve as a comparator to study the particulate form of HA (i.e., VLPs). Next, we used a 1,1′-dioctadecyl-3,3,3′,3′-tetramethylindodicarbocyanine perchlorate (DiD) dequenching assay^13^ and a panel of endocytosis inhibitors (Supplementary Table 1 and Supplementary Fig. 2c–e) to demonstrate that H1-VLPs were internalized primarily through CME and CIE with smaller contributions from macropinocytosis and, probably, phagocytosis (Fig. 2c). The prominent role of CME was further demonstrated by direct HA immunofluorescence in the presence of CME inhibitor chlorpromazine (Fig. 2d) and by co-localization of HA with fluorescently-labeled transferrin that is exclusively taken up by CME (Fig. 2e). Immunolabeling of clathrin and caveolin-1 in TEM images of MDMs exposed to H1-VLPs confirmed that the total endocytic vesicle pool included both clathrin-coated and caveolin-coated structures (Fig. 2f). Soluble H1 endocytosis was largely unaffected by the CIE inhibitor genistein but was greatly reduced by chlorpromazine (Fig. 2d). Internalized soluble HA was almost perfectly co-localized with transferrin (Fig. 2e).

In summary, MDMs exposed to H1-VLPs internalized much more HA compared to those pulsed with soluble H1. H1-VLP internalization occurred through multiple endocytic pathways including CME, CIE, macropinocytosis and, probably, phagocytosis while soluble H1 was internalized almost exclusively by CME. The diversity of H1-VLPs internalization mechanisms raised the possibility that antigen delivered in this form might experience different acidification and degradative environments, leading to a broader range of antigen processing and presentation pathways.^21^ It was therefore of interest to study endosomal trafficking and the intracellular fate of the two forms of HA.

### H1-VLPs are handled in two distinct endosomal pools: high- and low-degradative

**Fig. 3:**
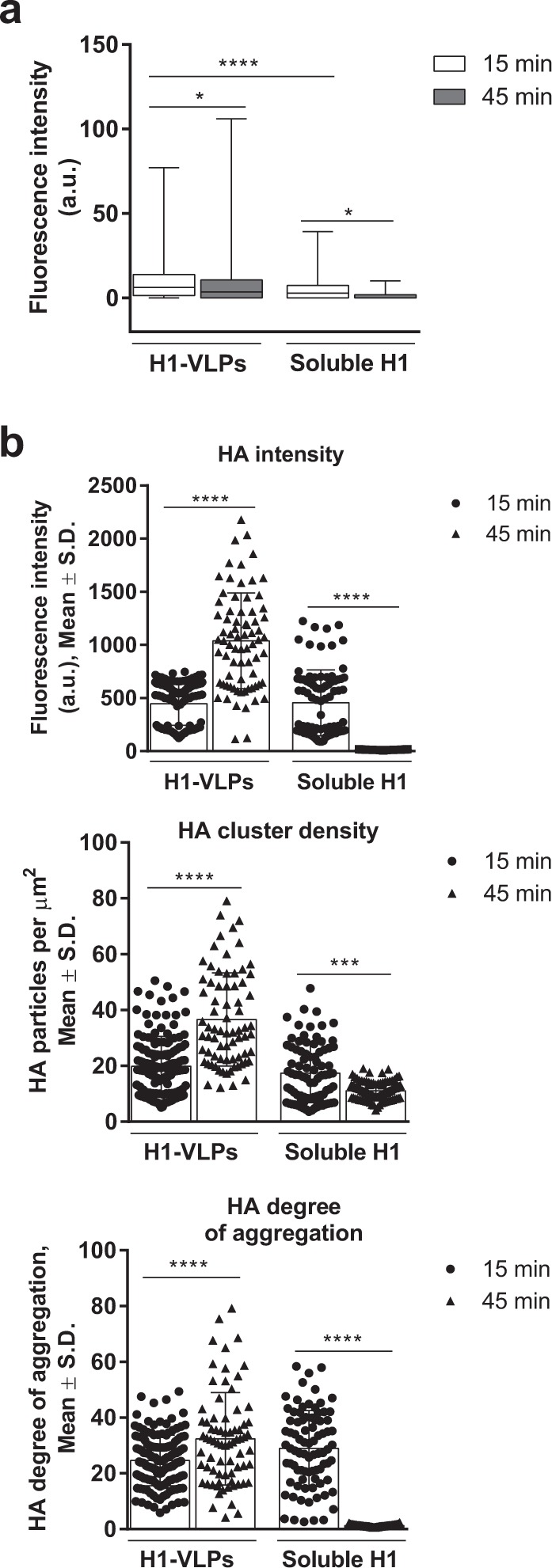
Intracellular HA distribution in human MDMs exposed to H1-VLPs or soluble H1. **a** HA internalization and degradation by MDMs pulsed with either H1-VLPs or soluble H1. The amount of internalized protein evaluated by the intensity of HA immunofluorescence per cell area on confocal microscopy images. Data from 9 experiments were analyzed. **b** Segmentation ICCS analysis of the HA endosomal distribution in MDMs pulsed (15 min) with either H1-VLPs or soluble H1 shows HA fluorescence intensity (top), cluster density (number of fluorescent particles per µm^2^—middle) and degree of HA aggregation (bottom). Based on 7 experiments. Bar graphs present mean ± standard deviation (S.D.); box and whisker plot presents the minimum, maximum, median, and 25th and 75th percentiles. *p < 0.05, ***p < 0.001, ****p < 0.0001 (one-way ANOVA followed by Tukey’s multiple comparisons post-test)

Both the amount of internalized HA and the degree of degradation over time varied with delivery form (Fig. 3a and Supplementary Fig. 3a). We used the intensity of HA fluorescence as a surrogate for protein degradation, assuming that the monoclonal antibody-binding epitope would be preserved in a low-degradative intracellular compartment (early-static or recycling endosomes). Conversely, the disappearance of the fluorescent signal would suggest trafficking to late endosomes/endolysosomes. Using confocal microscopy, the intensity of HA fluorescence in MDMs pulsed with soluble H1 dropped dramatically over 45 min (>90%) while the cells pulsed with H1-VLP retained ~75% of the HA signal, suggesting that a substantial portion of the internalized protein delivered by VLP had found its way into low-degradative cellular compartments. To more precisely define the fate of the internalized HA, we analyzed the confocal data using the fluorescence fluctuation method image cross-correlation spectroscopy (ICCS) with segmentation.^22,23^ We based the segmentation on HA-positive endosomes (Supplementary Fig. 3b) via automatic thresholding based on the fluorescence intensity. The intensity of HA fluorescence in the endosomes of VLP-pulsed cells increased more than two-fold between 15 and 45 min. The cluster density (number of HA particles per µm^2^) and the degree of HA aggregation also increased (Fig. 3b). In contrast, HA fluorescence intensity fell ~97% in the endosomes of MDMs pulsed with soluble H1 during this same time period. The cluster density and the degree of HA aggregation in these samples was greatly reduced as well, suggesting that the internalized soluble protein was almost completely degraded by 45 min.

Thus, exposure of MDMs to H1-VLPs resulted in rapid and substantial endocytosis. A large proportion of HA remained intact for at least 45 min after the VLP pulse. Moreover, increases in HA fluorescence intensity, cluster density, and degree of aggregation all suggested homotypic fusion of the HA-positive endosomes.^24^ The simultaneous reduction in overall HA fluorescence intensity per cell area argued for movement of some of the protein to high-degradative late endosomes, supporting a bidirectional trafficking model for the HA delivered by the VLPs. In contrast, the uptake of soluble H1 was less substantial at the outset (15 min) and the HA fluorescence had almost completely disappeared at 45 min. These observations prompted us to further characterize the endosomal compartments contributing to the complex handling of HA delivered on VLPs or as soluble protein.

### H1-VLPs move towards static early and/or recycling endosomes in human MDMs

**Fig. 4:**
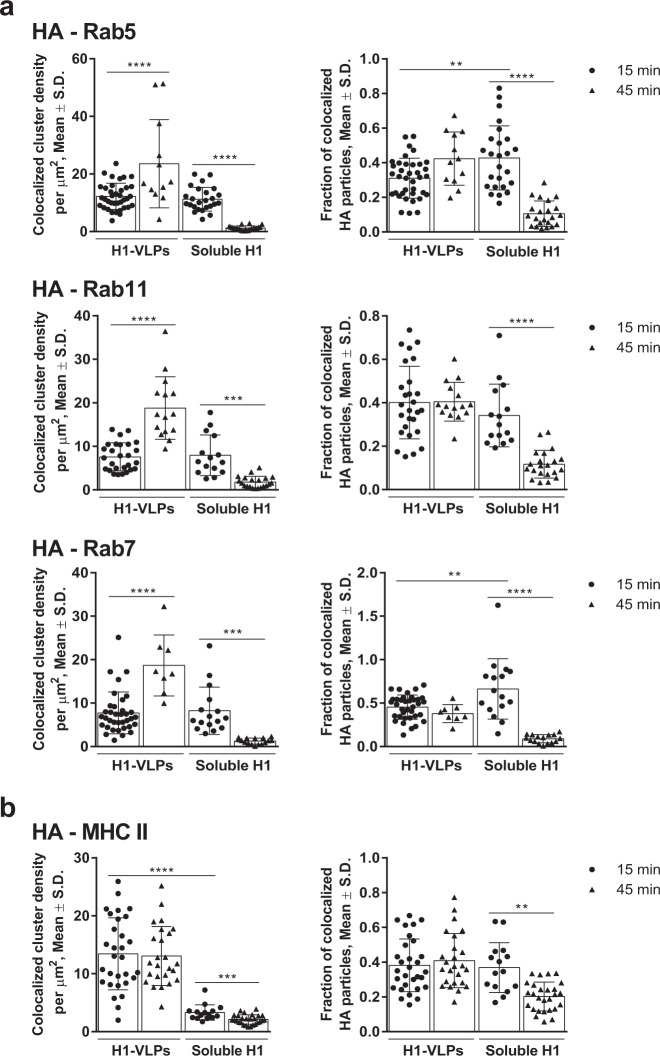
Segmentation ICCS analysis of HA colocalization with Rab proteins and MHC II. **a** HA colocalization (number of colocalized particles per µm^2^—left) and colocalized fraction of HA (right) with Rab5 (top), Rab11 (middle), and Rab7 (bottom) are presented. Based on 3 or more experiments for each condition. **b** HA colocalization (number of colocalized particles per µm^2^—left) and colocalized fraction of HA (right) with MHC II are presented. Based on three experiments. Bar graphs present mean ± standard deviation (S.D.). **p < 0.01, ***p < 0.001, ****p < 0.0001 (one-way ANOVA followed by Tukey’s multiple comparisons post-test)

Conventional colocalization analysis based on “per cell” image segmentation suggested that H1-VLPs preferentially track from early, Rab5-positive endosomes to low-degradative Rab11-positive recycling endosomes rather than to late Rab7-positive endosomes/endolysosomes (Supplementary Fig. 4a).^25^ Of note, in a significant minority of H1-VLP-exposed cells (~15%), we observed peripheral re-distribution (recycling) of undegraded HA towards the plasma membrane at 45 min (Supplementary Fig. 4b). Soluble H1 was partially co-localized with all three endosomal markers at 15 min but was undetectable in any endosomal compartment by 45 min, suggesting that the HA had been almost fully degraded. When the HA-positive endosomal compartment was characterized by segmentation ICCS analysis, there was a substantial increase in the HA colocalized cluster density (number of colocalized particles per µm^2^) with Rab5 and Rab11 markers at 45 min, suggesting protein retention in static early and/or recycling endosomes (Fig. 4a). Unexpectedly, we also observed an increase of HA–Rab7 colocalized cluster density, which may possibly be explained by Rab conversion of the slowly-maturing endosomes.^26^ The HA–MHC II colocalized cluster density was ~4-fold higher in the H1-VLP-pulsed MDMs compared to those treated with soluble H1 at 15 min, and did not change by 45 min (Fig. 4b), suggesting that VLPs facilitate HA delivery, at least partially, in high-degradative Rab7^+^ compartments that favor MHC II-restricted antigen presentation.^21,27^ The fraction of HA particles interacting with endosomal proteins remained unchanged between 15 and 45 min in the MDMs exposed to H1-VLPs (Fig. 4), suggesting that HA-enriched endosomes retained association with Rab proteins that regulate endosomal trafficking, cargo sorting, and organelle maturation.^28^

In summary, a large portion of the HA delivered on VLPs is retained in low-degradative endosomal compartments (static early and/or recycling endosomes) for at least 45 min, while the remainder follows the “classic” endosomal degradation pathway. In contrast, soluble H1 is mostly trafficked towards high-degradative intracellular compartments. These striking differences in the intracellular handling of HA raised questions about the possible immunological consequences of the two forms of antigen delivery.

### HA delivery in the form of VLPs favors antigen cross-presentation by human MDMs

**Fig. 5:**
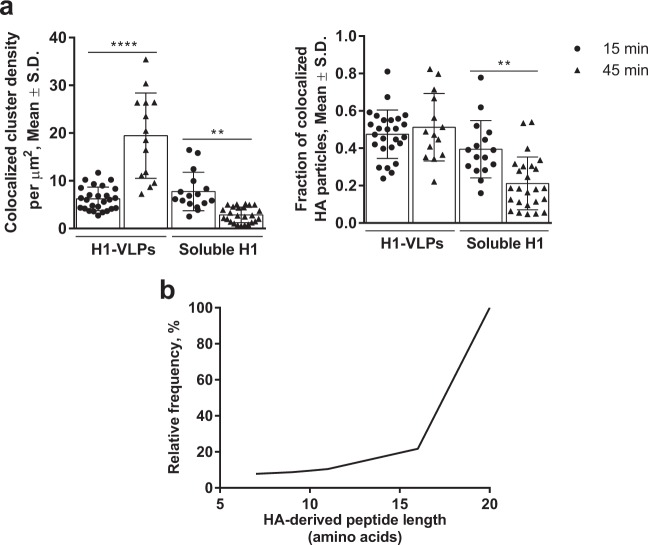
HA cross-presentation by human MDMs exposed to H1-VLPs. **a** Segmentation ICCS analysis of HA colocalization with MHC I. The colocalization (number of colocalized particles per µm^2^—left) and colocalized fraction of HA (right) with MHC I presented. Based on 3 experiments. Bar graphs present mean ± standard deviation (S.D.). **p < 0.01, ****p < 0.0001 (one-way ANOVA followed by Tukey’s multiple comparisons post-test). **b** Cumulative curve shows the distribution by length (number of amino acids) of the HA-derived peptides detected from H1-VLP-treated MDMs

A large portion of the intracellular MHC I pool resides in recycling Rab11a-positive endosomes that can support cross-presentation of phagocytosed antigens.^29^ It was therefore of interest to explore HA delivery into the MHC I-positive endosomal compartments following pulsing of MDMs with the different forms of HA. ICCS colocalization revealed a strong association between HA and MHC I molecules in MDMs pulsed with either H1-VLPs or soluble H1 at 15 min (Fig. 5a). By 45 min however, HA–MHC I colocalized cluster density had greatly increased in H1-VLP-exposed cells (213%) but fell by 63% in the MDMs pulsed with soluble HA. The fraction of interacting HA particles remained unchanged by 45 min in H1-VLP-exposed MDMs. Based on the assumption that prolonged retention of antigen in low-degradative (MHC I^+^, Rab11^+^) compartments favors cross-presentation,^30^ we immunoprecipitated MHC I–peptide complexes from lysates of MDMs that had been pulsed overnight with H1-VLPs or soluble H1 and analyzed the eluted peptides using high-resolution tandem MS. No confident HA-derived peptides were detected in the lysate of MDMs exposed to soluble H1. In contrast, 115 HA-derived MHC I-associated peptides were identified in MDMs exposed to the H1-VLPs (posterior error probability (PEP) score ≤0.01), contributing to an HA sequence coverage of 12–89% (Table 1 and Supplementary Table 2). Eight peptides were detected in more than one donor and the HA protein scores varied from 32 to 323. Among the 115 HA-derived peptides, 50 (43.5%) came from the globular head, 51 (44.3%) from the stem domain, and 14 (12.2%) from the transmembrane/intracellular portion of HA. Average protein quantity in cell lysates did not differ between HA treatment groups (2773.8 ± 595.2 µg/mL in VLP group and 2440.4 ± 468.1 µg/mL in soluble HA group; *p* = 0.74; Mann–Whitney test). The HA-derived peptides averaged 18 amino acids (AA) and only ~10% had an “optimal” length for MHC I loading (8–10 AA: Fig. 5b).^30^ This observation suggested that the lysates contained a mixture of optimally-trimmed peptides and immature peptides from the endoplasmic reticulum (ER)/endosome compartments still being processed and sorted for either presentation or degradation.^31^

**Table 1:** Mass spectrometry analysis of HA-derived peptides from H1-VLP or soluble H1-treated MDMs obtained from four donors

Parameters	Donor A	Donor B	Donor C	Donor D
**HA treatment**	**Soluble H1**	**–**	**Soluble H1**	**Soluble H1**
Total protein amount, µg	2397	–	1652	3272
Sequence coverage (%)	0	–	0	0
Protein score	0	–	0	0
# MS/MS spectra	0	–	0	0
# Peptides	0	–	0	0
**HA treatment**	**H1-VLPs**	**H1-VLPs**	**H1-VLPs**	**H1-VLPs**
Total protein amount, µg	2653	1444	2658	4340
Sequence coverage (%)	61	12	30	89
Protein score	32	75	152	323
# MS/MS spectra	29	11	16	84
# Peptides	25	10	14	66

## Discussion

As bridges between innate and adaptive immune responses, “professional” phagocytes such as macrophages are first-line defenders against invading pathogens.^32^ Among other activities, antigen uptake, processing, and display by these cells contribute to both the strength and the pattern of the immune response. In this in vitro work, we focused on human MDMs that phenotypically and functionally resemble the inflammatory-type macrophages^33^ implicated in orchestrating early responses to influenza virus infection.^34^ Our goal was to better understand how these cells handle influenza HA proteins when delivered either as a soluble protein (i.e., in the form of single molecules or small supramolecular complexes) or decorating the surface of ~100 nm plant-derived VLPs. HA was presented on the surface of VLPs in the form of trimers whereas soluble HA that lacks the transmembrane portion was delivered in the monomeric form.^19^ Since no difference was observed in the uptake of monovalent H1N1 influenza vaccine and recombinant soluble H1 (Fig. 2b and Supplementary Fig. 2b), we used the latter in these experiments in order to minimize possible biases introduced by the presence of influenza proteins other than HA, viral RNA, egg-derived components, detergent, and formaldehyde traces in the split virion vaccine.

Regardless of the production platform, VLPs are nanoparticles bearing viral antigens that resemble native virions in size and, in many cases, structure.^10^ VLPs are often highly immunogenic, but are non-infectious due to lack of the genetic material.^35–37^ Several very successful VLP vaccines based on recombinant antigens are in current use including those targeting hepatitis B virus and human papillomaviruses.^38^ VLPs can display a number of antigens with or without adjuvant molecules, and therefore have the potential to elicit broad immune responses.^36^ VLPs typically retain the native conformation of the antigens they display; they are organized in an ordered array and in a particulate form, all of which promote strong immune responses.^36,39^ Due to highly repetitive epitopes on their surface, VLPs can act as strong activators of antigen-presenting cells (APCs), facilitating internalization and presentation of antigens, resulting in strong induction of antibodies as well as virus-specific CD4^+^ and CD8^+^ T cells.^40,41^ A number of VLP-based vaccine candidates expressing influenza HA with or without other viral proteins are under development.^42–49^

VLPs can be produced in bacteria (*Escherichia coli*)^50^ or yeast,^51^ in insect,^52^ mammalian^53^ and plant cells^8^ and even in cell-free systems in vitro.^54^ Production in plants is attractive for several reasons,^55^ including low cost and scalability. Furthermore, expression of candidate vaccines in plants exploits their eukaryotic processing machinery, supporting appropriate post-translational modifications and assembly of antigens. Finally, plant-derived VLPs may have a significant safety advantage since the risk of contamination with human pathogens is extremely low.^56–58^ Plant-derived influenza vaccine candidate VLPs have been successfully produced by a number of academic and industrial groups.^8,59,60^

We have previously shown that plant-derived VLPs rapidly interact with human immune cells including B cells, monocytes, and dendritic cells^10,12,18^ and that early interactions with human MDMs (i.e., binding, internalization, entry into endosomes and fusion with endosomal membranes) are similar to what happens with wild-type influenza virions.^13^ The efficiency of the VLP–MDM interactions may largely be attributable to the particulate form of the VLPs bearing 30–50 HA trimers that stimulate rapid binding and internalization.^10,13^ In the current work, we tracked intracellular handling by MDMs at much higher resolution and demonstrated that a large proportion of the HA delivered on these VLPs enters static and recycling endosomal pathways leading to MHC I cross-presentation.

Many factors contribute to how APCs handle any given antigen including the dose and form of the antigen itself, the nature and activation state of the APCs as well as the microenvironment in which these processes occur. How the antigen first enters the cell can also strongly influence the outcome and APCs have many choices from “bulk” processes like phagocytosis and macropinocytosis or more controlled processes like CME or CIE. Antigens internalized through CME are usually delivered rapidly to maturing degradative endosomes that undergo quick acidification and fusion with lysosomes.^61^ The cleavage products of lysosomal proteases and peptidases are typically longer peptides (13–25 AA) appropriate for MHC II loading that leads to priming of CD4^+^ T cells and support of strong humoral responses.^27^ In our studies, H1-VLP-pulsed MDMs demonstrated substantially greater HA–MHC II colocalization than soluble H1-treated cells, suggesting that the VLPs may favor, at least in part, MHC II-restricted presentation of HA-derived peptides. We also observed nearly complete disappearance of soluble H1 immunofluorescence at 45 min, suggesting predominant trafficking towards the high-degradative endolysosomal compartment in human MDMs. Such handling is certainly consistent with the observation that split virion influenza vaccines typically elicit strong antibody responses but little-to-no priming of CD8^+^ T cells and only limited cell-mediated immunity against influenza.^6^

In contrast, CIE often leads to homotypic fusion of caveolin-coated endocytic vesicles and formation of large caveosomes that can retain non-degraded antigenic material for long periods of time.^62^ Although the mechanisms are not yet fully understood, both static early endosomes^63^ and non-acidified endosomes in the vacuolar pathway^30^ have been reported to support cross-presentation. Phagocytosis and macropinocytosis typically result in the internalization of large quantities of an antigenic material that may also favor cross-presentation and priming of CD8^+^ T cells.^30,64^ Although some of the HA-specific immunofluorescence was lost shortly after pulsing human MDMs with H1-VLPs, a substantial portion of the initial fluorescence was still detectable at 45 min and the ICCS analysis revealed striking increases in HA fluorescence intensity, cluster density, and aggregation in the endosomes. The trafficking of the HA delivered on the plant-derived VLPs therefore appeared to be bidirectional: with a small portion moving rapidly into the high-degradative late endosome/endolysosome pathway (similar to soluble HA) while a substantial amount was retained in low-degradative compartments. At 45 min, both immunostaining and ICCS analysis demonstrated increasing colocalization of the VLP HA with both Rab5^+^ (early static) and Rab11^+^ (recycling) low-degradative endosomes.^26,63^ Nair-Gupta et al. have reported that recycling Rab11a^+^ endosomes represent a major intracellular pool for MHC I molecules,^29^ and colocalization of the VLP-delivered HA with MHC I in these low-degradative compartments increased almost 3-fold between 15 and 45 min after the MDMs were pulsed. Thus, the 45 min exposure allowed us to detect endosomal degradation of the soluble HA and entry of the HA delivered by VLP into the static early/recycling endosomal compartments. Although the long-term kinetics of the disappearance of the VLP-delivered HA in the MDMs would be of some interest, it was not among the original goals of this work.

Although our imaging studies provided evidence for HA-VLP delivery into endosomal compartments that favor antigen cross-presentation, direct proof of HA processing by the human MDMs leading to the generation of MHC I-associated peptides was missing. MS-based immunopeptidome studies have historically required billions of cells to obtain sufficient numbers of MHC molecules for efficient detection of MHC-associated peptides.^65^ One consequence of this technical limitation is that most MS-based immunopeptidome work has focused on immortalized cell lines or animal cells.^66,67^ Recent improvements in MS technology combined with nano-flow chromatography now offer better sensitivity for the detection of MHC-associated peptides from relatively small numbers of cells. In our study, the use of a recently developed nanospray ion source with a constant flow of dopant gas permitted enhanced ionization and more efficient detection of MHC I peptides from only 3–10 million MDMs. The Maxis II mass spectrometer used in this study also has a unique hardware configuration that allows very high transmission of peptides into the collision cell enabling the detection of a wide range of peptides of various lengths.^68^ Our data unambiguously show that cross-presentation of HA peptides by human macrophages is possible when the HA is delivered by plant-derived VLPs. So far, MHC I-restricted presentation of influenza virus-derived peptides was thought to require infection or administration of the live attenuated vaccine, involving viral replication.^3,4^

Internal (structural) proteins of influenza viruses (i.e., nucleoprotein, polymerase basic protein 1, matrix protein 1) are thought the principle targets of the human CD8^+^ T cell response^3^ and only a limited number of influenza HA-derived MHC I-restricted peptides have been reported to date.^69,70^ We were therefore surprised to find 115 HA-derived peptides from the VLP-pulsed MDMs, 8 of which were identified in more than one donor (Supplementary Table 2). A substantial number of peptides arose from the stem region of HA molecule, raising the possibility that they may be relatively well-conserved across different viral strains with the potential to provide cross-protective immunity. Among the previously described 17 HA-derived human MHC I-restricted epitopes,^71^ 4 peptides (23%) were fully overlapping with up to three unique AA sequences identified in our study. Since we used unfractionated cell lysates containing MHC I molecules from the cell surface as well as those present in endosomes and the ER–Golgi compartment, it was not unexpected that many of the HA-derived peptides identified were longer than the 8–10 AA thought to be optimal for MHC I loading.^30^ It is likely that the longer peptides immunoprecipitated with MHC I were destined either for further trimming to achieve a better fit in the MHC I peptide binging groove or for degradation.^31,72^ The differences between donors in the number of MHC I-associated HA-derived peptides identified (i.e., fewer in donor B and more in donor D) could be attributable to individual and (possibly) racial, gender, or age characteristics. Together, the imaging and MS results presented herein collectively suggest that influenza HA delivery to human MDMs in the form of VLPs can result in cross-presentation. It is noteworthy that VLPs produced using other platforms have been previously shown to facilitate antigen cross-presentation.^73–75^

The potential for VLP-based vaccines to induce CD8^+^ T cells has been demonstrated in mice^18^ and studies are on-going to determine whether or not similar responses can be elicited in humans. Although conserved T cell epitopes from influenza core proteins have attracted the most attention to date,^3,70^ several human CD8^+^ epitopes have been identified in the HA proteins of both seasonal and avian influenza strains^76,77^ and our data suggest that many more may exist. A non-living vaccine that can induce strong antibody production as well as both CD4^+^ and CD8^+^ T cell responses might provide an added layer of protection against serious influenza virus infections compared to antibodies alone.^78^

## Methods

### Plant-made VLP bearing influenza hemagglutinin

The VLPs produced in *N. benthamiana* were kindly provided by Medicago Inc. (Quebec, QC). Plant-derived VLPs are readily available from the corresponding author upon reasonable request. The influenza HA protein was based on the sequence of A/California/07/2009 H1N1 virus. Recombinant soluble H1 protein (Immune Technology, New York, NY, Catalogue No. IT-003-SW12ΔTMp) of the same influenza strain and influenza A (H1N1) 2009 monovalent split vaccine (Sanofi Pasteur Inc., Swiftwater, PA) were used as comparators. The HA concentration in H1-VLP and soluble HA stocks was provided by the manufacturer based on the United States Food and Drug Administration (FDA)-validated enzyme-linked immunosorbent assay (ELISA) and an optical density (OD) 280 nm absorbance using absorbance coefficient established for the HA AA sequence, respectively.

### Gel electrophoresis and immunoblot analysis

H1-VLP and soluble H1 protein samples (2.5 and 5 µg per sample) were separated on Bis–Tris NuPAGE Gradient gel 4–12% (Life Technologies, Carlsbad, CA) under reducing conditions followed by Coomassie blue (Life Technologies) staining. For immunoblot analysis, 0.5 µg of each HA protein per sample was separated on the gel and transferred onto nitrocellulose membranes. Membranes were blocked in 5% milk–0.05% Tween-20 in phosphate buffer saline for 1 h at 22 °C and incubated overnight at 4 °C with rabbit polyclonal anti-H1 antibody (Catalogue No. IT-003-0011, Immune Technology, New York, NY) at a 1:250 dilution, or mouse monoclonal anti-H1 antibody (clone IVC102, Catalogue No. C86304M, Meridian Life Science, Memphis, TN) at a 1:2000 dilution. Membranes were incubated for 1 h at 22 °C with horseradish peroxidase-conjugated donkey anti-rabbit IgG (1:8000 dilution, Catalogue No. NA934V) or sheep anti-mouse IgG (1:20,000 dilution, Catalogue No. NA931V) antibodies, both from GE Healthcare Life Sciences, Uppsala, Sweden, respectively. Bands were developed using Super Signal West Pico Plus reagent (Pierce Biotechnology, Rockford, IL). Coomassie blue-stained or chemiluminescence bands were imaged using ChemiDoc™ XRS+ System (Bio-Rad Laboratories, Hercules, CA) and X-ray films. All blots derived from the same experiment and were processed in parallel. Full, un-cropped images of all blots, including full molecular weight markers, are provided in Supplementary Fig. 1a–c. The total protein concentrations in the H1-VLP and soluble H1 stocks was measured using Pierce™ BCA Protein Assay Kit (Pierce Biotechnology) according to the manufacturer’s instructions.

### Monocyte-derived macrophages (MDMs)

MDMs were differentiated from human peripheral blood mononuclear cells (PBMCs) isolated from healthy donors between the ages of 23 and 47. All studies with human cells were carried out with approval from the Research Ethics Committee of the McGill University Health Centre. Written informed consent was obtained from all donors prior to blood drawing. PBMCs were separated from whole blood by centrifugation using SepMate-50 tubes (STEMCELL, Vancouver, BC). Monocytes were isolated by negative selection using magnetic microbeads according to the manufacturer’s instructions (EasySep Human Monocyte Enrichment Kit, STEMCELL). Monocytes were cultured in Roswell Park Memorial Institute (RPMI)-1640 with 50 IU/mL penicillin, 50 µg/mL streptomycin, and 10 mM 4-(2-hydroxyethyl)-1-piperazineethanesulfonic acid (HEPES) (medium) supplemented with 10% fetal bovine serum (FBS, all from Wisent, Saint-Jean-Baptiste, QC) and 20 ng/mL recombinant human macrophage colony-stimulating factor (Gibco, Frederick, MD) for 7 days.

### VLPs endocytosis assessment based on fluorescence dequenching

VLPs were stained with DiD (Thermo Fisher Scientific, Eugene, OR) at 20 µg/mL for 30 min at 22 °C, and then purified from free dye using gel filtration columns (PD MiniTrap G-25, GE Healthcare, Buckinghamshire, UK). MDMs were detached from plastic plate surface using Accutase Cell Detachment Solution (BioLegend, San Diego, CA) and plated on 96-well Nunclon Delta black flat-bottom plates (Thermo Fisher Scientific, Roskilde, Denmark) at 5 × 10^4^ cells/well. The following day, MDMs were exposed to DiD-labeled VLPs (HA concentration 15.0 µg/mL) at 4 °C for 1 h. Endocytosis inhibitors were applied in ice-cold medium supplemented with 10% FBS: dynasore hydrate 50 µM, genistein 200 µM, amiloride hydrochloride 1 mM, cytochalasin D 4 µM (all from Sigma-Aldrich, St. Louis, MO). DiD fluorescence was measured with pre-heated (37 °C) spectrophotometer (Infinite 200 PRO, Tecan, Männedorf, Switzerland) at 15-min intervals over 2 h. Fusion efficiency was determined following the addition of Triton X-100 (Sigma-Aldrich) to each well (final concentration 1%) to obtain full DiD dequenching.

### Immunostaining and confocal microscopy

MDMs were exposed to H1-VLPs, soluble H1 or influenza A (H1N1) 2009 monovalent split vaccine at concentrations 15 or 5 µg/mL (by HA content) in 5% CO_2_ incubator at 37 °C for 5, 10, 15, or 45 min. Endocytosis inhibitor chlorpromazine hydrochloride (10 µg/mL, Sigma-Aldrich) or genistein (200 µM) was applied 30 min prior to adding H1-VLPs or soluble H1, and the duration of HA exposure was 15 min. Transferrin (human) CF568 conjugate (Biotium, Fremont, CA) was mixed with either H1-VLPs or soluble H1 (both transferrin and HA concentrations 15 µg/mL), and the mixture was applied to the MDMs for 15 min (5% CO_2_, 37 °C). In the pulse-exposure experiments, MDMs were exposed to H1-VLPs or soluble H1 for 15 min, and then the supernatant was replaced by medium and kept for another 30 min at 5% CO_2_ and 37 °C. MDMs were fixed with 4% methanol-free formaldehyde (Thermo Fisher Scientific, Rockford, IL), blocked and permeabilized with 5% goat and 5% donkey serum (both from EMD Millipore Corporation, Darmstadt, Germany) in 0.3% Triton X-100. HA immunostaining was performed using mouse anti-H1 antibody (diluted 1:200, clone IVC102, Catalogue No. C86304M, Meridian Life Science, Memphis, TN). Endosomal proteins were visualized with rabbit anti-Rab5 (diluted 1:200, clone C8B1, Catalogue No. 3547), anti-Rab7 (diluted 1:100, clone D95F2, Catalogue No. 9367), anti-Rab11 (diluted 1:100, clone D4F5, Catalogue No. 5589) antibodies (all from Cell Signaling Technology, Danvers, MA), anti-HLA-DPB1 (diluted 1:100, clone EPR11226, Catalogue No. ab157210, Abcam, Cambridge, MA) or anti-HLA-A antibody (diluted 1:250, clone EP1395Y, Catalogue No. GTX61514, GeneTex, Irvine, CA). Secondary donkey anti-mouse Alexa Fluor 488 (diluted 1:1000, Catalogue No. A21202) and goat anti-rabbit Alexa Fluor 647 antibodies (diluted 1:1000, Catalogue No. A21245, both from Thermo Fisher Scientific, Rockford, IL) were applied for 1 h at 22 °C. NucBlue Live ReadyProbes Reagent—4′,6-diamidino-2-phenylindole (DAPI, Thermo Fisher Scientific) used to stain cell nuclei. A laser scanning confocal microscope (Zeiss LSM780, RI-MUHC Molecular Imaging Core Facility, Montreal, QC) was used in all imaging experiments. Fluorescence intensity after cell-based segmentation of the images was quantified using ImageJ software.^79^

### Quantitative image analysis

The fluorescence fluctuation analysis method ICCS was applied to evaluate the HA endosomal distribution and the degree of HA colocalization with endosomal proteins. In brief, ICCS measures molecular concentrations and interaction fractions based on correlation analysis of fluorescence fluctuations detected from imaged biomolecules as a function of space across an image. Automatic thresholding of fluorescence intensity via Otsu’s method was applied to identify the high intensity HA-positive endosomal area in images. The negative control (no HA) samples were not suitable for ICCS since they did not display the HA fluorescence needed to segment the HA-positive endosomes for analysis. The number of HA particles per µm^2^, the HA fluorescence intensity and the aggregation state of HA particles in the HA-positive endosomal area per cell can be calculated from the spatial autocorrelation function of the fluorescence intensity fluctuations in an image from a single detection channel. Particles formed by endosomal proteins of interest (Rab5, Rab7, Rab11, MHC I, and MHC II) were also identified.

To evaluate the colocalization between HA and specific endosomal proteins, we calculated the spatial cross-correlation function from the fluorescence intensity fluctuations between images recorded in two different wavelength detections channels (expressed as (i) number of colocalized HA particles per µm^2^ and (ii) fraction of interacting HA particles) in two-color images.

### Transmission electron microscopy (TEM)

MDMs were exposed to H1-VLPs or soluble H1 at concentration 15 µg/mL (by HA content) in 5% CO_2_ incubator at 37 °C for 5 or 15 min before fixation with 2.5% glutaraldehyde (EMS Inc., Hatfield, PA). Samples for nanogold immuno-labeling were initially fixed with 2% methanol-free formaldehyde (15 min at 22 °C) and permeabilized/blocked with 0.2% Triton X-100 plus 1% goat serum for 5 min on ice. Primary mouse anti-H1 (diluted 1:200, clone IVC102, Catalogue No. C86304M, Meridian Life Science), or rabbit anti-clathrin (diluted 1:50, clone D3C6, Catalogue No. 4796) or anti-caveolin-1 (diluted 1:200, clone D46G3, Catalogue No. 3267) antibodies (both from Cell Signaling Technology) were applied overnight at 4 °C. Secondary Alexa Fluor 647—FluoroNanogold goat anti-mouse (2 µg/mL, Catalogue No. 7501) or Nanogold goat anti-rabbit (diluted 1:50, Catalogue No. 2004) antibodies, respectively (both from Nanoprobes, Yaphank, NY) were applied in 1% non-fat dried milk, and then cells were fixed with 2.5% glutaraldehyde. Samples were washed with 0.1 M sodium cacodylate (EMS Inc.). Cells were silver-enhanced for 30 s using HQ Silver enhancement kit (Nanoprobes). Post-fixation was done in 1% osmium tetroxide (EMS Inc.) containing potassium ferrocyanide (Fisher Scientific, Pittsburgh, PA). Cells were dehydrated with 0–100% ethanol and progressively embedded with EPON 812 REPLACEMENT™ kit (Mecalab Ltd., Montreal, QC). The samples were sectioned and imaged with a Tecnai T12 microscope (FEI Inc., Hillsboro, OR).

### Mass spectrometry analysis

MDMs were exposed to either H1-VLPs or soluble H1 at HA concentration 15 µg/mL in 5% CO_2_ incubator at 37 °C for 16 h. Cells were lysed (1 h, 4 °C) with a buffer containing 4% NP-40 Surfact-Amps™ (Thermo Fisher Scientific, Rockford, IL), 50 mM Tris–HCl (pH 7.0), 150 mM NaCl and protease inhibitors (Pierce™ Protease Inhibitor Mini Tablets, Thermo Fisher Scientific). Lysate total protein concentration was determined using Pierce™ BCA Protein Assay (Thermo Fisher Scientific). MHC I–peptide complexes were immunoprecipitated using Dynabeads™ protein G immunoprecipitation kit (Thermo Fisher Scientific) and anti-human HLA-A,B,C antibody (clone W6/32, Catalogue No. 311402, BioLegend, 10 µg per 500 µL lysate, 45 min, 4 °C). Dynabeads™-antibody–antigen complexes were washed three times, transferred into clean tubes, and the peptides were eluted with 10% acetic acid (70 °C, 15 min). The immunoprecipitation eluate was cleaned on a C18 solid-phase extraction Macro Spin column (Harvard Apparatus, Holliston, MA) using water and methanol following manufacturer’s instructions, and evaporated to dryness under vacuum (37 °C, 120 min). Samples were reconstituted in 0.1% formic acid (60 μL) and injected (20 μL) onto a Maxis II (Bruker, Billerica, MA) high-resolution quadrupole-time of flight tandem mass spectrometer equipped with a Dionex UltiMate 3000 (Thermo Fisher Scientific, Waltham, MA) ultra-high-performance liquid chromatography (UHPLC) system using an Acclaim PepMap 300 RSLC C18 2 μm 100 Å 150 × 0.075 mm UHPLC column (Thermo Fisher Scientific) with water (A) and acetonitrile (B) both containing 0.1% formic acid at a flow rate of 0.3 μL/min (50 °C). Elution gradient started at 5% B, was held for 3 min, then increased to 35% at 73 min, 55% at 90 min, and 80% at 95 min. MS spectra were acquired at *m/z* 400–2200 and MS/MS spectra were recorded at *m/z* 150–2200 using collision-induced dissociation (CID) activation in Auto MS/MS mode with a collision energy of 21–55 eV depending on precursor ion *m/z* value and charge state (*z*). Ions with *z* = 2–5 were preferred whereas singly charged ions were excluded. Redundant ions were also excluded for 2 min. Acquisition time was 0.5 s for MS and 0.06–0.25 s for each MS/MS scan depending on precursor ion signal intensity, with a total cycle time of 3.0 s. CaptiveSpray (Bruker) nanospray ionization source operated in positive mode with a capillary voltage of 1.8 kV. To enhance ionization, a continuous flow of nitrogen and vaporized acetonitrile (as dopant) was injected into the ion source during the analysis using a nanoBooster module (Bruker). Nitrogen (99.5% pure) was used as dry gas (150 °C) at a flow rate of 3.0 L/min. Samples were analyzed in duplicate.

### Analysis of proteomics data

Mass spectra were imported into the MaxQuant software^80^ and searched against an in-house influenza A virus (A/California/07/2009(H1N1)) hemagglutinin FASTA file including six UniProt-TrEMBL identifiers: C3W627, C3W5X2, I6T4Z8, R9RVT8, U3M8B4, U3M8F8. An unspecific search was conducted for peptides with a length of 5–20 residues at 0.01 false-discovery rate (FDR). Methionine oxidation and N-terminal acetylation were defined as variable modifications. A PEP threshold of 0.01 was set for all MaxQuant searches. To account for LC retention shifts, the “match between runs” option was enabled with a match time window of 0.7 min and an alignment time window of 20 min. Only hits with a protein score >30 were accepted.

### Statistical analysis

Statistical analysis was performed using GraphPad Prism 6.0 software. One-way analysis of variance (ANOVA) followed by Tukey’s multiple comparisons post-test or Mann–Whitney test was used to examine the differences between samples. *p*-values < 0.05 were considered statistically significant.

## Supplementary information

Supplementary information.

## Data Availability

The datasets generated and/or analyzed during the current study are available from the corresponding author. The MS proteomics data have been deposited to the ProteomeXchange Consortium via the PRoteomics IDEntifications (PRIDE) partner repository^81^ with the dataset identifier PXD010519.

## Supplementary information

**Supplementary information** accompanies the paper on the *npj Vaccines* website (10.1038/s41541-019-0111-y).

## Code availability

Custom MATLAB code used for ICCS is available from the corresponding author upon reasonable request.
