# Supplementary material for: Plant-derived virus-like particle vaccines drive cross-presentation of influenza A hemagglutinin peptides by human monocyte-derived macrophages
Source: NPJ Vaccines. 2019 May 15;4:17. doi: 10.1038/s41541-019-0111-y (PMC6520342; doi:10.1038/s41541-019-0111-y)
Supplement: Supplementary file 1 — Supplementary information. [file 41541_2019_111_MOESM1_ESM.pdf]

SUPPLEMENTAL INFORMATION

Title:

Plant-derived virus-like particle vaccines drive cross-presentation of influenza A hemagglutinin peptides by human monocyte-derived macrophages

Authors:

Alexander I. Makarkov, Makan Golizeh, Elizabeth Ruiz-Lancheros, Angelica A. Gopal, Ian N. Costas-Cancelas, Sabrina Chierzi, Stephane Pillet, Nathalie Charland, Nathalie Landry, Isabelle Rouiller, Paul W. Wiseman, Momar Ndao, Brian J. Ward

Supplemental Methods

Electron microscopy (EM)

For negative stain preparation, the VLP samples were diluted with PBS pH 7.4 – 100 µg/mL based on estimated influenza hemagglutinin (HA) content. 5 µL of the diluted VLP solution was placed for 45 sec on 200 Hex grids (EMS Inc., Hatfield, PA), formerly carbon coated and glow-discharged. The grids were washed twice with 5µL of distilled water for 45 sec followed by 45 sec incubation with 2% uranyl acetate (EMS Inc.). Excess fluid was removed by blotting with a filter paper and samples were left to air dry. The grids were imaged commonly on a Tecnai T12 (FEI Inc., Hillsboro, OR). The MDM samples processing for EM is described in the manuscript, Methods section.

Endocytosis inhibitors screening

A number of endocytosis inhibitors were screened based on their effect on virus-like particles (VLPs) internalization measured by 1,1'-dioctadecyl-3,3,3',3'-tetramethylindodicarbocyanine perchlorate (DiD - Thermo Fisher Scientific, Eugene, OR) fluorescence dequenching upon VLP fusion with cell membranes. H1-VLPs (influenza HA based on the sequence of A/California/07/2009 H1N1 virus, Medicago Inc., Quebec, QC) were labelled with DiD (see Methods section of the manuscript). B10R cell culture (an immortalized murine bone marrow-derived macrophage cell line) was maintained in RPMI-1640 with 50 IU/mL penicillin, 50 µg/mL streptomycin and 10 mM HEPES (medium) supplemented with 10% fetal bovine serum (FBS, all from Wisent, Saint-Jean-Baptiste, QC) until reaching cell confluency. B10R cells were detached from plastic flask surface using 0.25% trypsin/2.21 mM EDTA in HBSS (Wisent) and plated on 96-well Nunclon Delta black flat-bottom plates (Thermo Fisher Scientific, Roskilde, Denmark) at 5 x 10^4 cells per well. The following day, B10R cells in

triplicate wells were exposed to DiD-labelled VLPs in the medium (HA concentration 15.0 µg/mL); plates were kept at 4°C for one hour and then washed with ice-cold medium twice. Endocytosis inhibitors were applied in ice-cold medium supplemented with 10% FBS (100 µL/well): dynasore hydrate (50 µM), chlorpromazine hydrochloride (10 µg/mL), sucrose (0.45 M), pitstop 2 (25 µM), genistein (200 µM), filipin III from Streptomyces filipinensis (5 µg/mL), amiloride hydrochloride (1 mM), cytochalasin D (4 µM) (all from Sigma-Aldrich, St. Louis, MO). Pitstop 2 was also tested at the same concentration in serum-free medium. The plates were placed into a pre-heated (37°C) spectrophotometer (Infinite 200 PRO, Tecan, Männedorf, Switzerland), and DiD fluorescence was measured at 15-min intervals over 2 h. Fusion efficiency (%) was determined following addition of Triton X-100 (Sigma-Aldrich) to each well (final concentration 1%) to obtain full DiD dequenching.

Toxicity assessment of endocytosis inhibitors

B10R cell culture was maintained in the medium supplemented with 10% FBS until reaching cell confluency. B10R cells were detached from plastic flask surface using 0.25% trypsin/2.21 mM EDTA in HBSS and plated on 96-well Nunclon Delta black flat-bottom plates at 5 x 10^4 cells per well. The following day, endocytosis inhibitors (see the endocytosis inhibitors screening section and the Supplemental Table 1) in the medium supplemented with 10% FBS were applied to B10R cells in triplicate wells in a volume 100 µL/well (5% CO_2, 37°C for 2 h). Chlorpromazine hydrochloride was applied at concentrations 10 and 100 µg/mL. Pitstop 2 was also tested in serum-free medium. Triton X-100 0.01% solution served as a positive control. The effects of endocytosis inhibitors on B10R cells viability were evaluated in parallel experiments with the CytoTox-ONE™ homogeneous membrane integrity assay and the CellTiter-Glo® 2.0 assay (both from Promega, Madison, WI). Cell membrane integrity was assessed by lactate

dehydrogenase (LDH) release. CytoTox-ONE™ reagent (100 µL/well) was applied for 10 min at RT. Stop solution (50µl) was then added to each well, and the fluorescence was measured on Infinite 200 PRO spectrophotometer at excitation and emission wavelengths 560 and 600 nm, respectively. The results were reported as a % of the maximum LDH release caused by adding lysis solution to the control wells. The effects of endocytosis inhibitors on metabolically active cells were quantitated by the amount of ATP with CellTiter-Glo® luminescent cell viability assay. CellTiter-Glo® 2.0 reagent (100 µl/well) was applied for 10 min at RT. Luciferase luminescence was measured on Infinite 200 PRO spectrophotometer. The results were reported as a % of ATP level reduction compared to control wells unexposed to any endocytosis inhibitor.

Conventional image analysis

Confocal microscopy images were analyzed with ImageJ software[1] for the purpose of evaluation the fluorescence intensity of immunolabelled HA or fluorophore-conjugated transferrin, or for the assessment of colocalization of HA with endosomal markers Rab5, Rab7 and Rab11.[2] In brief, to analyze the HA or transferrin fluorescence intensity per cell area we identified cellular boundaries on the brightfield channel, and used them to establish the regions of interest (ROIs) on the fluorescent channel(s). Then we determined the background fluorescence intensity in each experiment by averaging the values obtained from cells in the control sample (for HA: monocyte-derived macrophages (MDMs) unexposed to HA but stained with anti-HA primary and fluorescent secondary antibody; for transferrin: MDMs unexposed to transferrin). The average background fluorescence intensity was subtracted from the fluorescence values measured from HA or transferrin-exposed cells. For "cell-based" colocalization analysis, both "green" and "red" channels were denoised with ImageJ PureDenoise plugin,[3] then image background was subtracted with the "rolling ball" algorithm.[4]

HA colocalization with endosomal markers was determined within cell boundaries-defined ROIs (see above) using Colocalization Threshold plugin;[5] Costes thresholding approach was applied.[6] Pearson correlation coefficient R, Pearson coefficient for pixels whose intensity falls above a threshold value R (>t), and Manders above threshold colocalization coefficients tM1 and tM2 were analyzed.[2,7,8]

Statistical analysis

Statistical analysis was performed using GraphPad Prism 6.0 software. One-way analysis of variance (ANOVA) followed by Tukey's multiple comparisons post-test, two-way ANOVA followed by Tukey's multiple comparisons post-test or Mann-Whitney test were used to examine the differences between samples. P values < 0.05 were considered statistically significant.

Supplemental tables

Supplemental Table 1

Endocytosis inhibitors used in screening experiments and their suggested mechanism of action

**Inhibitor of endocytosis**	**Tested concentration / condition**	**Suggested mechanism of action**	**Toxicity**
Dynamin-dependent endocytosis			
Dynasore	50 µM	Non-competitive and reversible inhibitor of GTPase activity of dynamin.[9] Dynasore suppresses both CME and CIE.[10,11]	Non-toxic
Clathrin-mediated endocytosis (CME)			
Chlorpromazine	10 – 100 µg/mL	Not well understood. It has been suggested that chlorpromazine causes AP-2 and clathrin relocation from plasma membrane to endosomal membranes and therefore depletes AP-2 and clathrin from the plasma membrane and prevents clathrin-coated endocytic vesicles formation.[12] Chlorpromazine probably affects dynamin activity.[13]	Non-toxic at 10 µg/mL. Toxicity observed at higher concentrations
Hyperosmotic sucrose	0.45 M	Not well understood. It has been suggested that hyperosmolarity leads to trapping clathrin in “microcages” and depleting it from plasma membrane.[14]	Greatly reduced ATP level in cells
Pitstop 2	25 µM (applied in serum-free or 10% FBS supplemented medium)	Not well understood. Pitstop 2 was developed as cell-permeable selective CME inhibitor.[15] However, later pitstop 2 has been shown to potently inhibit CIE.[16,17]	Toxic in serum-free medium (recommended use due to sequestering by serum albumins)
Clathrin-Independent Endocytosis			
Genistein	200 µM	Tyrosine-kinase inhibitor.[18] Phosphorylation of tyrosine at caveolin-1 is the prerequisite for pinching off caveolar vesicles from plasma membrane.[19]	Non-toxic
Filipin III	5 µg/mL	Cholesterol depleting and lipid-raft disrupting agent.[20]	Moderate cytotoxicity and

			massive ATP level reduction.
		Macropinocytosis	
Amiloride	1 mM	Inhibition of Na^+/H^+ exchange leads to lowering submembranous pH and preventing Rac1 and Cdc42 signaling that is essential for actin remodeling.[21]	Non-toxic
		Phagocytosis / macropinocytosis	
Cytochalasin D	4 μM	Blocking of actin polymerization, disassembly of actin cytoskeleton.[22,23]	Non-toxic

Supplemental Table 2

HA-derived peptides associated with major histocompatibility complex (MHC) I identified in MDM lysates using high-resolution tandem mass spectrometry

##	Sequence *	Length	Mass	Start position	End position	Charge state	PEP	MS/MS Count	Sample
1	AILVVLLYTFATANADTLCI	20	2124.1541	3	22	2	0.01	2	D
2	VVLLYTFATANADTLCIGYH	20	2184.0925	6	25	3	0.01	1	D
3	LYTFATANADTLCIGYHANN	20	2171.9946	9	28	3	0.01	1	D
4	LCIGYHA	7	775.3687	20	26	2	0.01	1	B
5	LCIGYHANNSTDTVDTVLEK	20	2192.0419	20	39	3	0.01	1	D
6	CIGYHAN	7	776.3276	21	27	2	0.01	1	B
**7, 8**	**GYHANNSTDTVDTVLEKNVT**	**20**	**2177.0237**	**23**	**42**	**3**	**0.01**	**3(A), 2(D)**	**A, D**
**9, 10**	**DTVLEKNVTVTHSVNLLEDK**	**20**	**2253.1852**	**34**	**53**	**3(A), 2(D)**	**0.01**	**1**	**A, D**
11	LEDKHNGKLCKLRGVAPLHL	20	2240.2576	50	69	3	0.01	1	D
12	GKLCKLRGVAPLHLGK	16	1689.0236	56	71	3	0.01	1	C
13	LRGVAPLHLGKCNIAGWILG	20	2087.1826	61	80	2	0.01	1	A
14	PLHLGKC	7	766.4160	66	72	2	0.01	1	B
15	LGNPECESLSTASSWSYIVE	20	2170.9729	79	98	3	0.01	1	D
16	LSTASSWSY	9	1000.4502	87	95	2	0.00	1	A
17	TASSWSYIVETPSSDNGTCY	20	2166.9052	89	108	3	0.01	1	D
18	SWSYIVETPSSDNGTCYPGD	20	2176.8895	92	111	2	0.01	2	A
19	VETPSSDNGTCYPGDFIDYE	20	2207.8841	97	116	3	0.01	1	D
20	ETPSSDNGTCYPGDFIDYEE	20	2237.8583	98	117	3	0.01	1	A
21	PSSDNGT	7	676.2664	100	106	2	0.01	1	B
22	PSSDNGTCYPGDFIDYEELR	20	2276.9532	100	119	3	0.01	1	D
23	CYPGDFI	7	813.3367	107	113	2	0.01	1	B
24	YEELREQLSSVSSFERFEIF	20	2494.2016	115	134	3	0.01	1	A
25	VSSFERFEIFPKTSSWPNHD	20	2409.1390	125	144	3	0.01	1	D

26	RFEIFPKTSSWPNHDSNKGV	20	2345.1553	130	149	2	0.01	1	D
27	SSWPNHDSNKGVTAACPHAG	20	2034.8966	138	157	3	0.01	2	D
28	SNKGVTAACPHAGAKSFYKN	20	2050.0054	145	164	3	0.01	1	D
29	KGVTAACPHAGAKSFYKNLI	20	2075.0986	147	166	3	0.01	1	D
30	HAGAKSF	7	716.3606	155	161	2	0.01	2	B
31	AKSFYKNLIWLVKKGNSYPK	20	2383.3416	158	177	3	0.01	2	D
32	NLIWLVKKGNSYPKLSKSYI	20	2350.3413	164	183	3	0.01	2	D
33	IWLVKKGNSYPKLSKS	16	1847.0669	166	181	2	0.01	1	C
34	WLVKKGNSYPKLSKSYINDK	20	2367.2951	167	186	3	0.01	1	D
35	VKKGNSYPKLSKSYINDKGK	20	2253.2481	169	188	3	0.01	1	A
36	KLSKSYINDKGKEVLVLWGI	20	2289.3097	177	196	3	0.01	2	D
37	SKSYINDKGKEVLVLWGIHH	20	2322.2485	179	198	3	0.01	1	D
38	NDKGKEVLVLWGIHHPPTSA	20	2197.1644	184	203	3	0.01	1	A
39	DKGKEVLVLWGIHHPSTSAD	20	2188.1277	185	204	3	0.01	1	A
40	EVLVLWGIHHPSTSADQQSL	20	2216.1226	189	208	3	0.01	1	D
41	LWGIHHPSTSADQQSLYQNA	20	2252.0610	193	212	3	0.01	1	D
42	PSTSADQ	7	704.2977	199	205	2	0.01	1	B
43	LYQNADAYVFVGSSRY	16	1851.8792	208	223	3	0.01	1	C
44	YVFVGSSRYSKKFKPEIAIR	20	2374.3161	215	234	3	0.01	2	D
45	VFVGSSRYSKKFKPEIAIRP	20	2308.3056	216	235	3	0.01	3	D
46	SSRYSKKFKPEIAIRPKVRD	20	2404.3703	220	239	3	0.01	1	D
47	SRYSKKFKPEIAIRPKVRDR	20	2473.4394	221	240	3	0.01	1	A
48	RYSKKFKPEIAIRPKVRDRE	20	2515.4499	222	241	3	0.01	1	D
49	SKKFKPEIAIRPKVRD	16	1911.1418	224	239	3	0.01	3	C
50	KFKPEIAIRPKVRDREGRMN	20	2439.3645	226	245	3	0.01	1	D
51	KPEIAIRPKVRDQEGR	16	1891.0752	228	243	3	0.01	1	C
52	IRPKVRDREGRMNYYWTLVE	20	2580.3383	233	252	3	0.01	1	D
53	RDREGRMNYYWTLVEPGDKI	20	2497.2172	238	257	3	0.01	1	D
54	PGDKITFEATG	11	1134.5557	253	263	3	0.00	1	B
55	ATGNLVVPRYAFAMERNAGS	20	2123.0582	261	280	2	0.01	1	A

**56, 57**	**YAFAMERNAGSGIIISDTPV**	**20**	**2111.0357**	**270**	**289**	**2**	**0.01**	**1**	**A, D**
58	FAMERNAGSGIIISDTPVHD	20	2129.0212	272	291	2	0.01	1	D
59	AGSGIIISDTPVHDCNTTCQ	20	2030.9037	278	297	3	0.01	1	D
60	SGIIISDTPVHDCNTTCQTP	20	2100.9456	280	299	3	0.01	1	A
**61, 62**	**SDTPVHDCNTTCQTPKGAIN**	**20**	**2100.9205**	**285**	**304**	**2(A), 3(D)**	**0.01**	**1**	**A, D**
63	TPVHDCNTTCQTPKGAINTS	20	2086.9412	287	306	3	0.01	1	A
64	KGAINTSLPFQNIHPI	16	1748.9574	300	315	2	0.01	1	C
65	GAINTSLPFQNIHPITIGKC	20	2123.1197	301	320	3	0.01	1	A
66	PITIGKCPKYVKSTKL	16	1775.0379	314	329	3	0.01	1	C
67	YVKSTKLRLATGLRNI	16	1832.0996	323	338	3	0.01	1	C
68	YVKSTKLRLATGLRNIPSIQ	20	2257.3270	323	342	3	0.01	1	D
69	TKLRLATGLRNIPSIQ	16	1780.0683	327	342	3	0.01	1	C
70	RLATGLRNIP	10	1109.6669	330	339	2	0.00	1	C
71	LATGLRNIPSIQSRGLFGAI	20	2083.1902	331	350	3	0.01	1	D
72	RNIPSIQSRGLFGAIAGFIE	20	2145.1695	336	355	3	0.01	1	D
73	IPSIQSRGLFGAIAGFIEGG	20	1989.0684	338	357	2	0.01	1	D
74	SIQSRGLFGAIAGFIEGGWT	20	2066.0585	340	359	3	0.01	1	D
**75, 76**	**LFGAIAGFIEGGWTGMVDGW**	**20**	**2082.9873**	**346**	**365**	**3**	**0.01**	**1(A), 6(D)**	**A, D**
77	IAGFIEGGWTGMVDGWYGYH	20	2214.9833	350	369	3	0.01	1	D
78	GFIEGGWTGMVDGWYGYHHQ	20	2295.9796	352	371	3	0.01	1	D
79	GYHHQNEQGSGYAADLKSTQ	20	2189.9726	367	386	3	0.01	1	A
80	STQNAIDEITNKVNSVIEKM	20	2233.1260	384	403	3	0.01	1	D
81	FTAVGKEFNHLEKRIENLNK	20	2386.2757	407	426	3	0.01	1	D
82	EFNHLEKRIENLNKKVDDGF	20	2444.2448	413	432	3	0.01	1	D
83	FNHLEKRIENLNKKVDDGFL	20	2428.2863	414	433	3	0.01	1	D
84	LEKRIEN	7	900.5029	417	423	3	0.01	1	B
**85, 86**	**NKKVDDGFLDIWTYNAELLV**	**20**	**2352.2002**	**425**	**444**	**3**	**0.01**	**1**	**A, D**
87	VDDGFLDIWTYNAELLVLLE	20	2337.1780	428	447	3	0.01	1	D
88	DDGFLDIWTYNAELLVLLEN	20	2352.1525	429	448	2;3	0.01	2	D
89	DIWTYNAELLVLLENERTLD	20	2419.2271	434	453	3	0.01	1	D

90	LVLLENERTLDYHDSN	16	1929.9432	443	458	3	0.01	1	C
**91, 92**	**LLENERTLDYHDSNVKNLYE**	**20**	**2464.1870**	**445**	**464**	**2**	**0.01**	**1**	**A, D**
93	LDYHDSNVKNLYEKVRSQLK	20	2448.2761	452	471	3	0.01	1	D
94	SNVKNLYEKVRSQLKNNAKE	20	2361.2765	457	476	2	0.01	1	D
95	VKNLYEKVRSQLKNNA	16	1903.0639	459	474	2	0.01	1	C
96	RSQLKNNAKEIGNGCFEFYH	20	2354.1226	467	486	3	0.01	1	D
97	NNAKEIGNGCFEFYHKCDNT	20	2302.9736	472	491	3	0.01	2	D
98	GNGCFEFYHKCDNTCMESVK	20	2310.9166	478	497	2	0.01	1	D
99	EFYHKCDNTCMESVKNGTYD	20	2382.9555	483	502	2	0.01	1	D
100	CDNTCMESVKNGTYDYPKYS	20	2316.9337	488	507	3	0.01	1	A
101	TCMESVK	7	796.3459	491	497	2	0.01	1	B
102	KNGTYDYPKYSEEAKLNREE	20	2433.1448	497	516	3	0.01	1	D
103	REEIDGVKLESTRIYQILAI	20	2345.2955	514	533	2	0.01	1	D
104	DGVKLESTRIYQILAIYSTV	20	2268.2365	518	537	2	0.01	1	D
105	STRIYQILAIYSTVASSLVL	20	2197.2358	524	543	3	0.01	1	D
106	RIYQILAIYSTVASSL	16	1797.0036	526	541	2	0.01	1	C
**107, 108**	**QILAIYSTVASSLVLVVSLG**	**20**	**2032.1820**	**529**	**548**	**3**	**0.01**	**3(A), 2(D)**	**A, D**
109	AIYSTVASSLVLVVSLGAIS	20	1949.1085	532	551	3	0.01	1	D
110	IYSTVASSLVLVVSLGAISF	20	2025.1398	533	552	3	0.01	1	D
111	STVASSLVLVVSLGAISFWM	20	2066.1122	535	554	2	0.01	1	D
112	VASSLVLVVSLGAISFWMCS	20	2068.0737	537	556	3	0.01	1	A
113	VASSLVLVVSLGAISF	16	1560.9127	537	552	3	0.01	1	C
114	LVLVVSLGAISFWMCSNGSL	20	2095.0846	541	560	3	0.01	1	A
115	SLGAISFWMCSNGSLQCRIC	20	2174.9734	546	565	3	0.01	2	D

*** sequences found in more than one sample highlighted in bold font**

Supplemental Figures

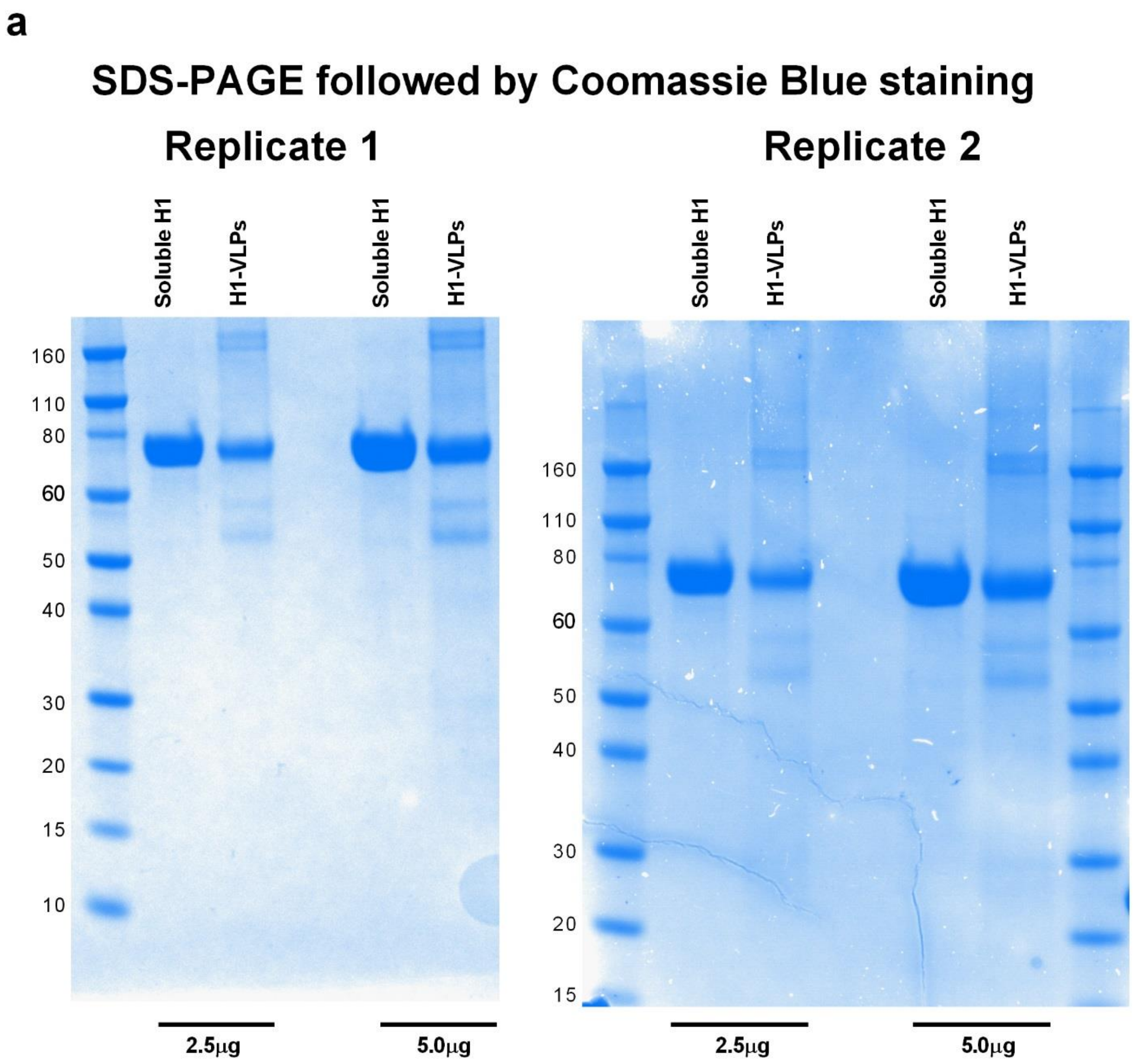

(continued on next page)

b

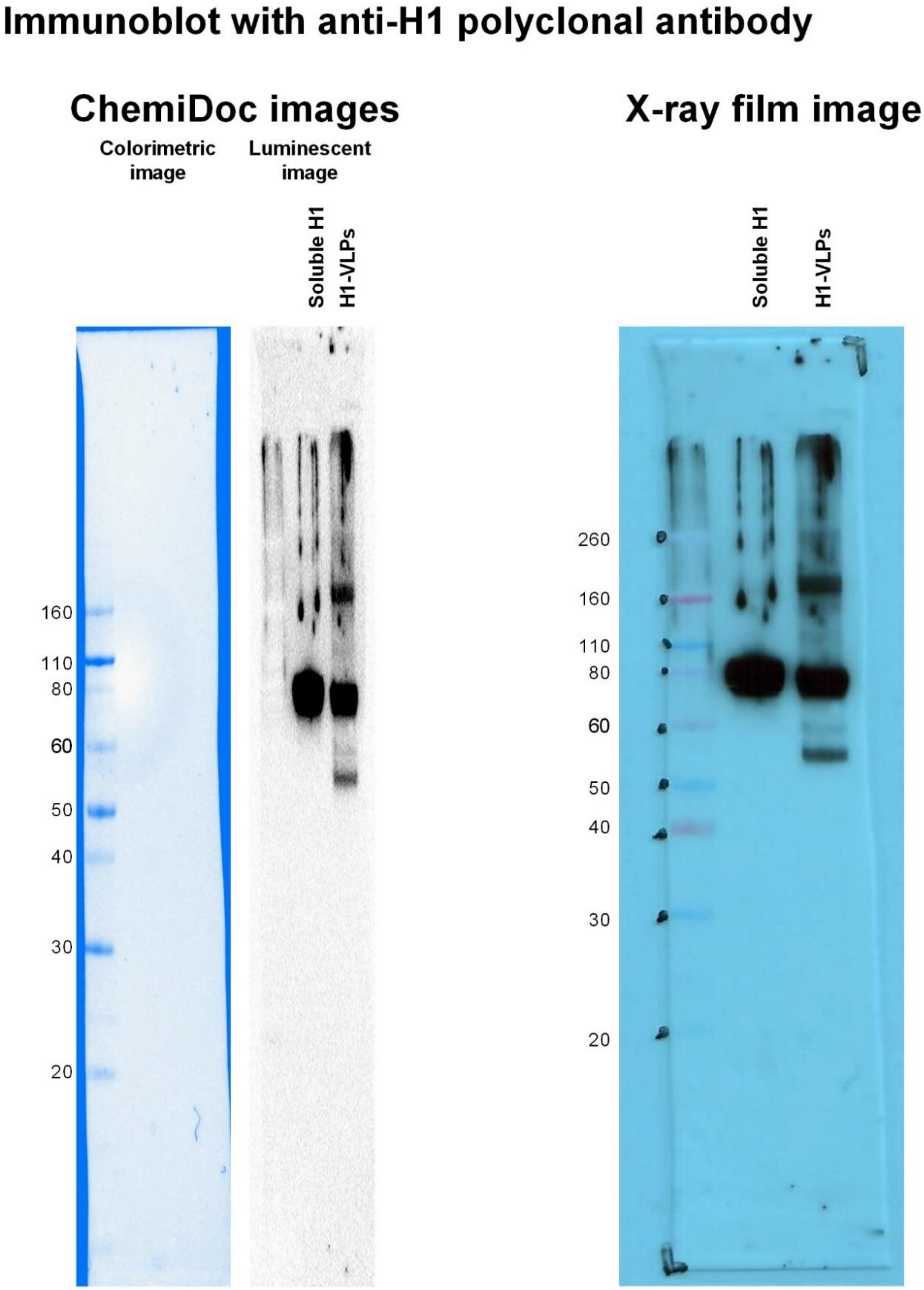

(continued on next page)

c

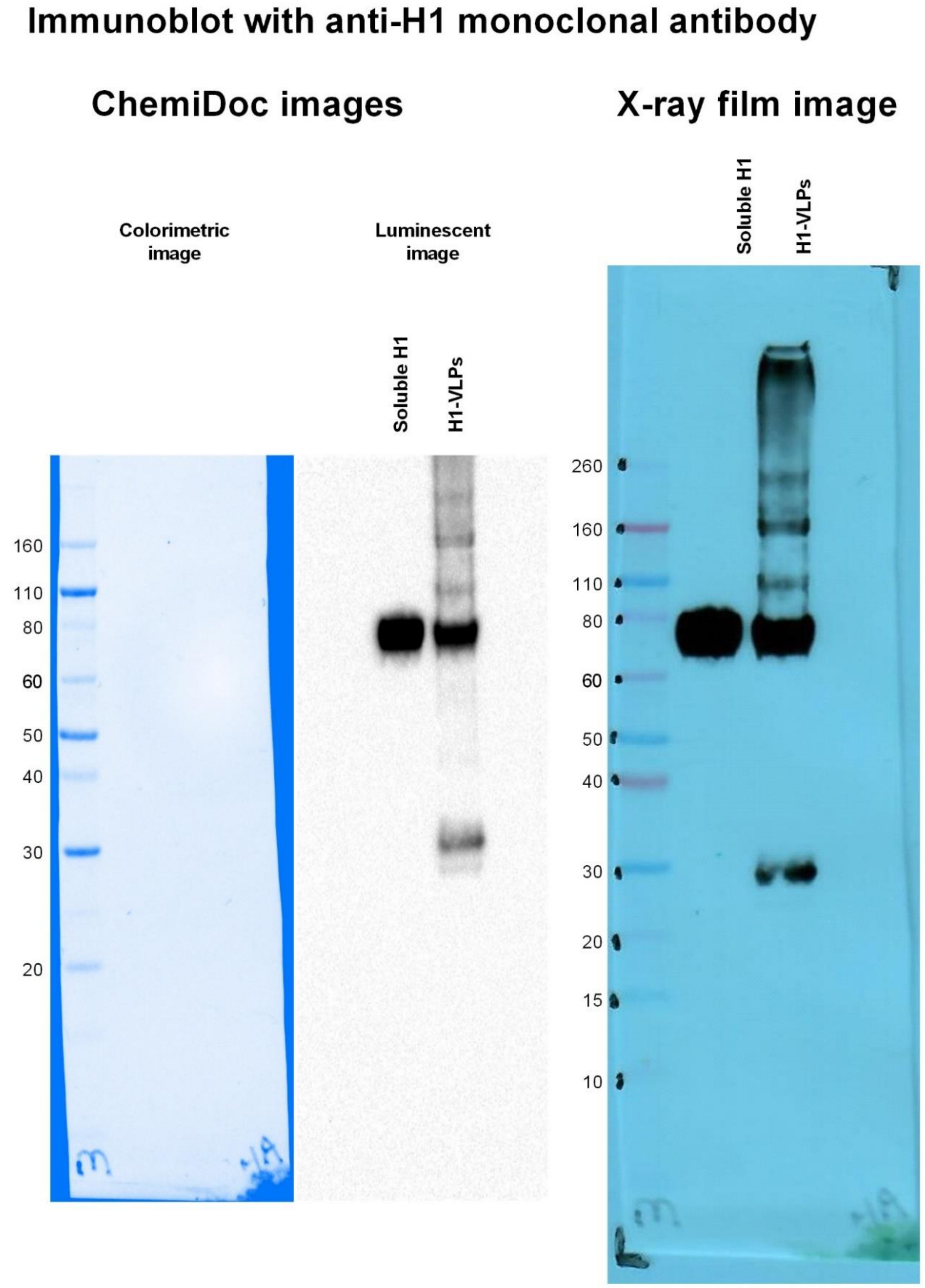

(continued on next page)

d

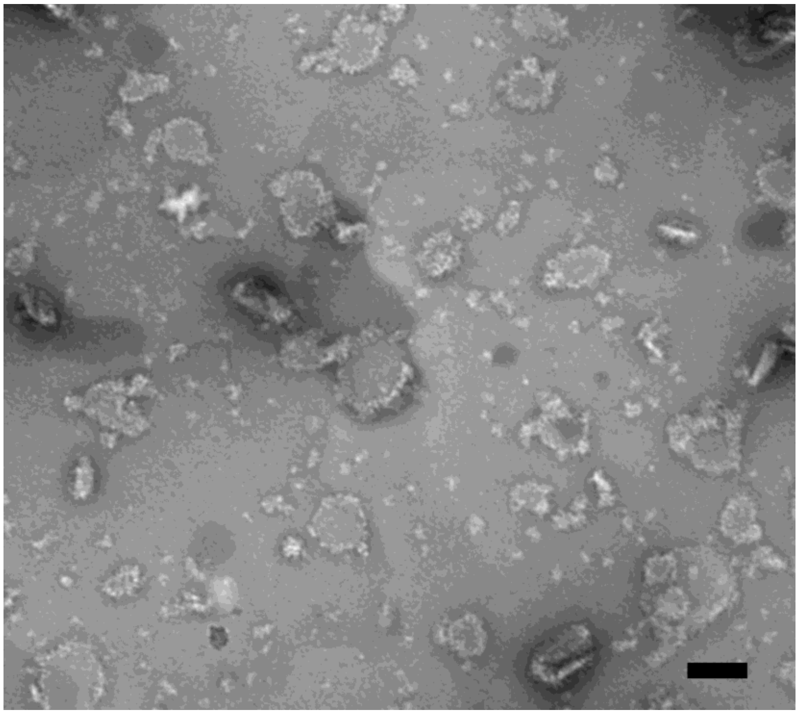

e

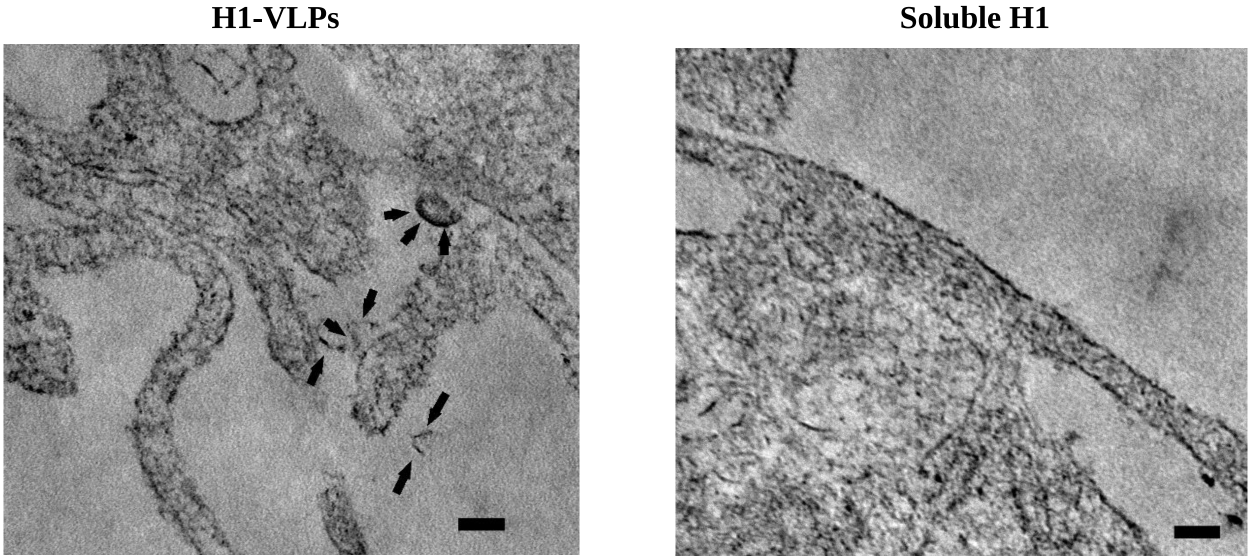

Supplemental Figure 1. Gel Electrophoresis, Immunoblot and EM characterization of plant-derived H1-VLPs and soluble recombinant H1 (comparator).

(a-c) Full, un-cropped images of SDS-PAGE with Coomassi blue staining and immunoblots,

(legend continued on next page)

including full molecular weight markers. **(a)** Two representative examples of SDS-PAGE gels stained with Coomassie blue. The H1-VLPs and soluble recombinant H1 (comparator) were applied at 2.5 and 5.0 µg per sample (by HA content). The predominant protein observed in both samples corresponded to the molecular weight of monomeric H1 (~ 75 kDa). No additional bands suggesting HA cleavage or degradation products were found in the soluble H1 sample (comparator). The H1-VLPs sample had a small number of less prominent bands at ~160 kDa (probably HA dimers) and at ~55 kDa (probably HA1 subunit). Immunoblot images of H1-VLPs and soluble H1 (both applied at 0.5 µg per sample by HA content) with polyclonal (**b**) or monoclonal (**c**) anti-H1 antibodies. In both cases, images were acquired using the ChemiDoc™ XRS+ System (ChemiDoc) with molecular weight markers (left) or immunostained for H1 (middle). X-ray images of the same membranes are also presented (right). The predominant protein observed in both samples was again monomeric H1 (~ 75 kDa) with no additional bands suggesting HA cleavage or degradation products in the soluble H1 sample (comparator). For the VLP sample, a small number of fainter bands were seen on both immunoblots as well as the X-ray images: ~160 kDa (HA dimers) and ~55 kDa (HA1 subunit). On some blots immunostained with the monoclonal anti-H1, faint bands were present at ~240 kDa (likely HA trimmers) and at 30 kDA (HA2 subunit). The low evident band at ~ 105 kDa probably represents the uncleaved H1-HA2 subunit complexes. **(d)** Negative stain EM of H1-VLP sample. Representative image shows pleomorphic particles ~ 100 nm average diameter with spikes on their surface. Scale bar – 100 nm. **(e)** Representative images of human MDMs exposed to H1-VLPs (left) or soluble H1 (right) at HA concentration 15 µg/mL. Arrows indicate VLP-like structures on the surface on VLP-treated MDM. Scale bar – 100 nm.

a

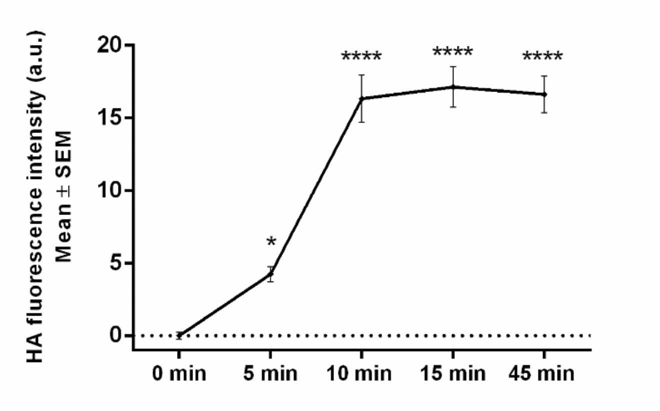

b

H1-VLPs, 15 µg/mL　　Split H1N1 vaccine, 15 µg/mL

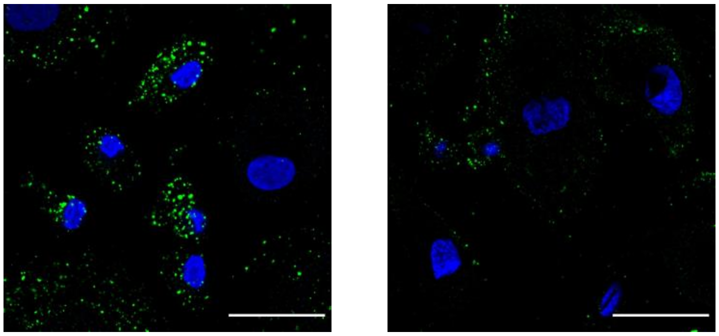

H1-VLPs, 5 µg/mL　　Split H1N1 vaccine, 5 µg/mL

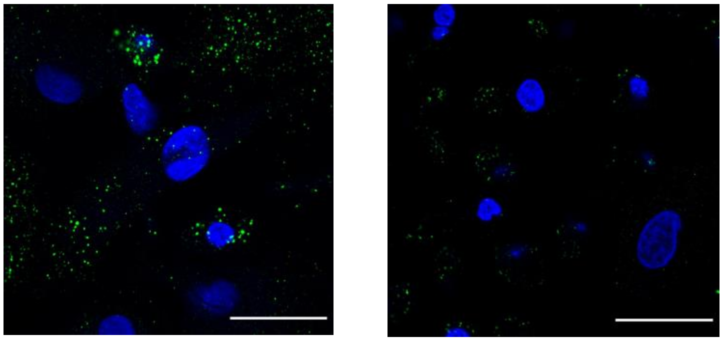

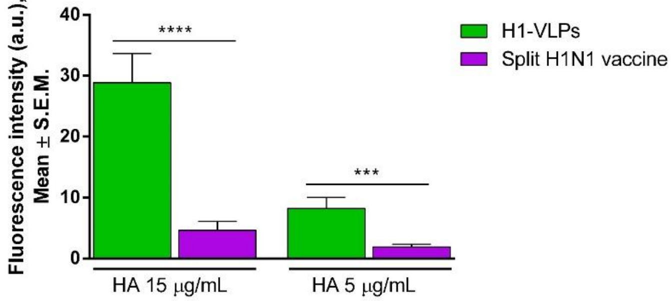

(continued on next page)

c

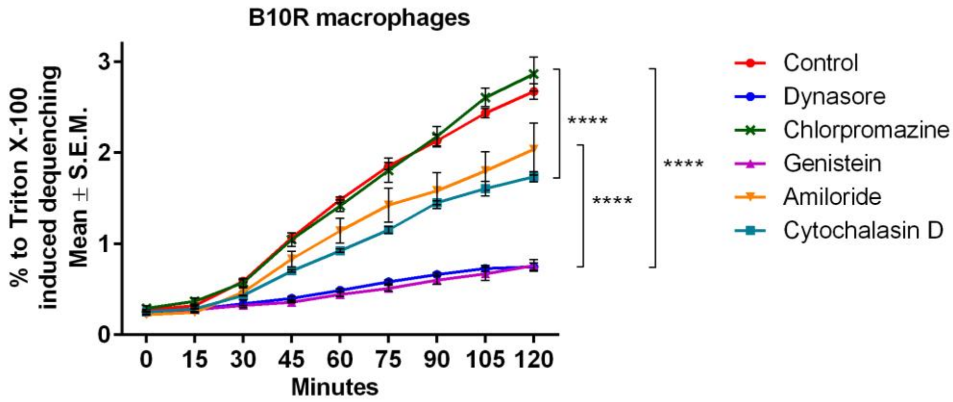

d

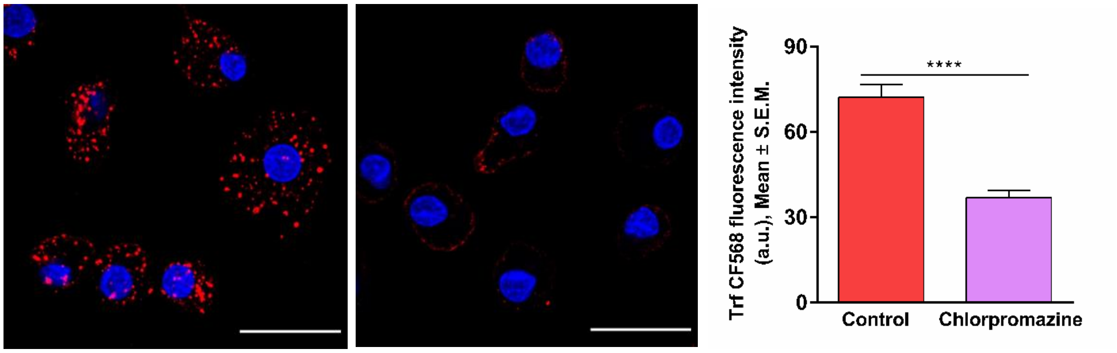

e

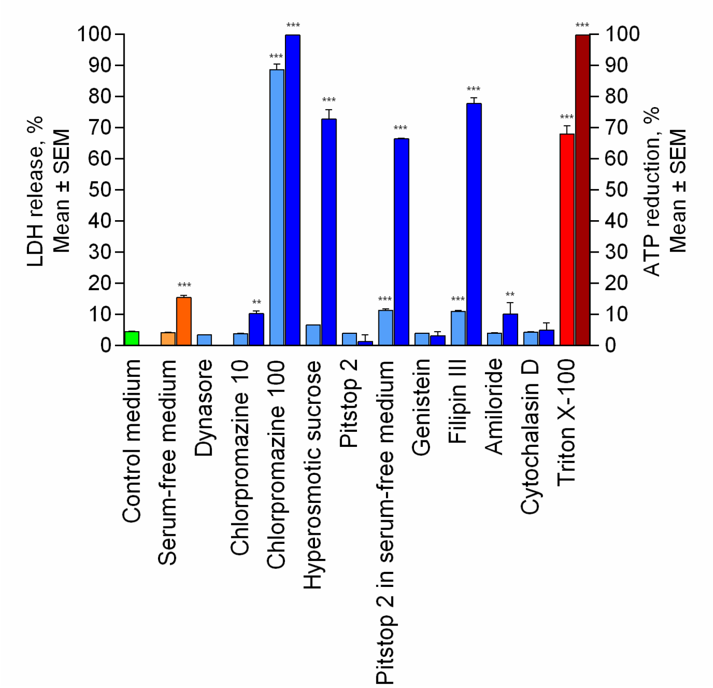

(legend on next page)

(legend on next page)

Supplemental Figure 2. Endocytic pathways in human and murine macrophages.

(a) Time-course of HA internalization by human MDMs. The amount of internalized protein was evaluated by the intensity of HA immunofluorescence per cell area on confocal microscopy images. The fluorescence intensity increased by 10 min and then remained at the same level up to 45 min. Based on three experiments. **(b)** Comparison of HA internalization by human MDMs exposed to either H1-VLPs or influenza A (H1N1) 2009 monovalent split vaccine for 30 min. Representative images of MDMs treated with H1-VLPs or monovalent H1N1 split vaccine at HA concentration 15 µg/mL (top) or 5 µg/mL (middle). Green: fluorescently labeled HA, blue: nuclei stained with DAPI. The amount of internalized protein was evaluated by the intensity of HA immunofluorescence per cell area on confocal microscopy images (bottom). Scale bar – 25 µm. **(c)** Effects of selected endocytosis inhibitors on DiD fluorescence dequenching by murine B10R macrophages loaded with DiD-labeled H1-VLPs (n=3). Chlorpromazine (10 µg/mL) did not affect DiD fluorescence. **(d)** Chlorpromazine (10 µg/mL) reduced transferrin (Trf) uptake by human MDMs. Representative images of the control sample treated with fluorescently-labelled transferrin (left), cell exposed to transferrin in the presence of chlorpromazine (middle) and the analysis of transference fluorescence intensities (right) are presented; based on three experiments. Red: transferrin conjugated with CF568 fluorophore, blue: nuclei stained with DAPI. Scale bar – 25 µm. **(e)** Cytotoxic effects of endocytosis inhibitors on murine B10R macrophages assessed by LDH release (left Y axis) and ATP level reduction (right Y axis) (n=3). Chlorpromazine hydrochloride (100 µg/mL), sucrose (0.45 M), pitstop 2 (25 µM) applied in serum-free medium, and filipin III from *Streptomyces filipinensis* (5 µg/mL) caused prominent LDH release and/or ATP level reduction in B10R macrophages. Mean ± Standard Error of the Mean (S.E.M.) presented on all graphs; * $p<0.05$, ** $p<0.01$, *** $p<0.001$, **** $p<0.0001$ (a, b,

e - one-way ANOVA followed by Tukey's multiple comparisons post-test; c - two-way ANOVA followed by Tukey's multiple comparisons post-test; d - Mann-Whitney test).

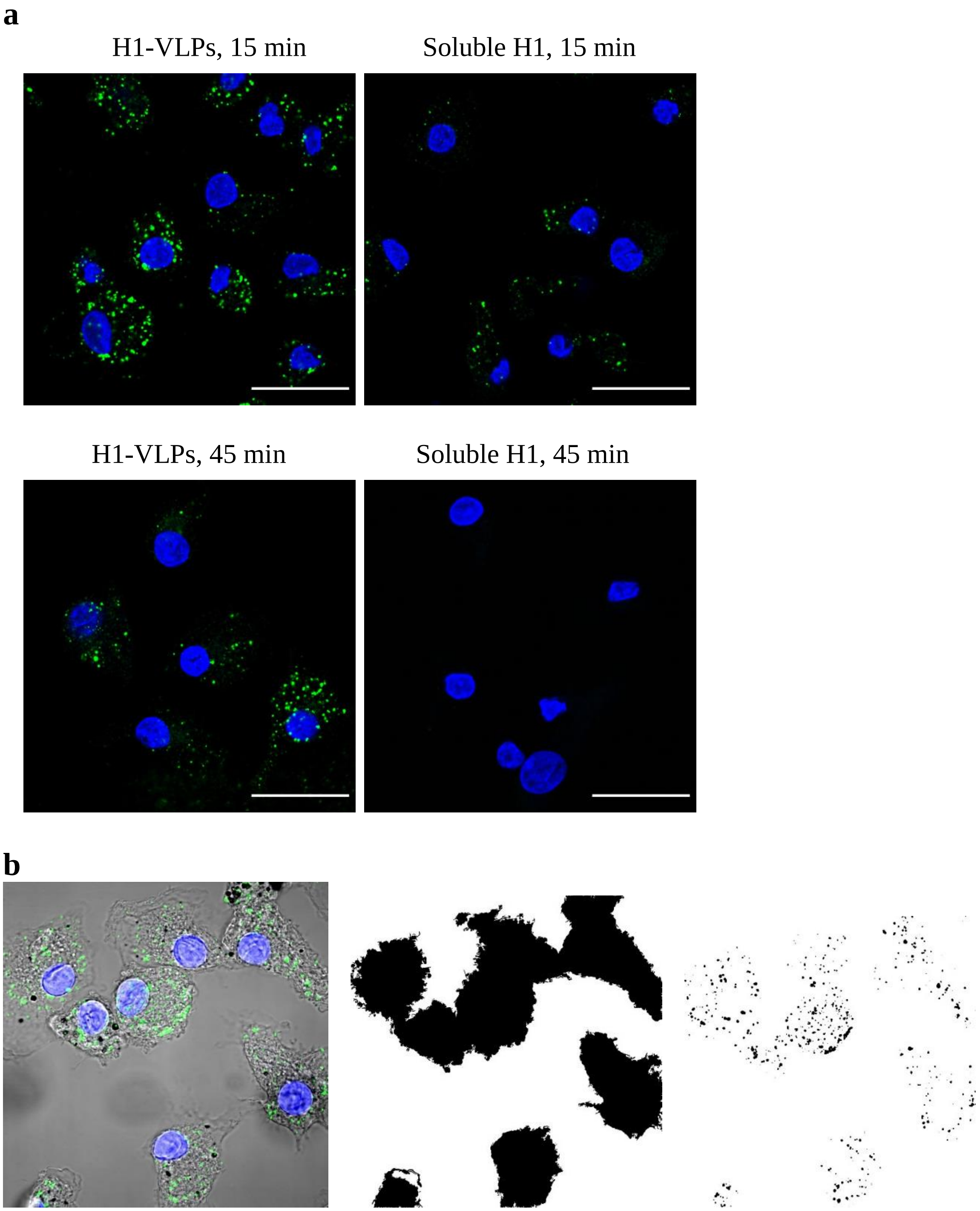

Supplemental Figure 3. HA internalization by human MDMs exposed to H1-VLPs or soluble H1.

(a) Representative images of MDMs pulsed (15 min) with H1-VLPs or soluble H1 at 15 min and

(legend continued on next page)

45 min. Green: fluorescently labeled HA, blue: nuclei stained with DAPI. Scale bar – 25 µm. **(b)** Examples of image segmentation strategies. Left - bright-field image of MDMs with internalized HA (green) and nuclei stained with DAPI (blue). Image segmentation based on identification of cell boundaries – entire cell area (middle). Image segmentation based on detecting HA-positive endosomes (right).

a

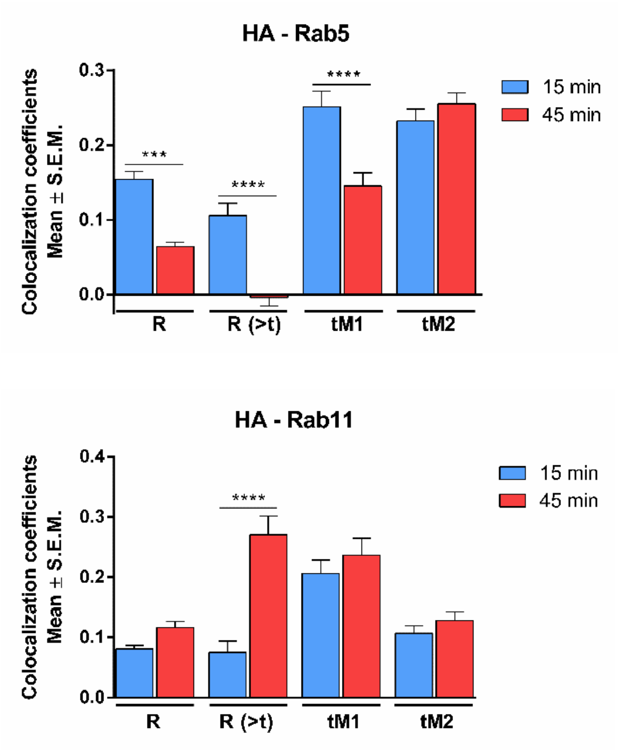

b

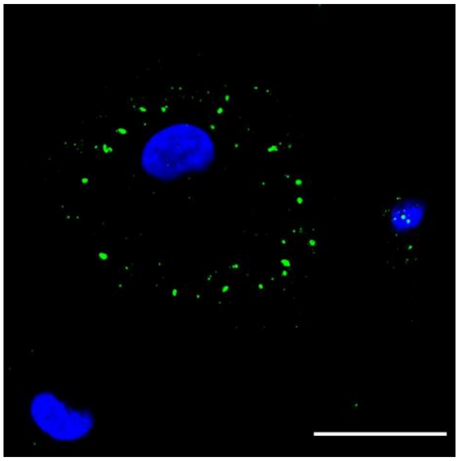

Supplemental Figure 4. Endosomal trafficking of HA in MDMs pulsed (15 min) with H1-VLPs or soluble H1.

(a) Conventional analysis of HA colocalization with Rab5, Rab7 or Rab11. Based on three or more experiments for each condition. Mean ± Standard Error of the Mean (S.E.M.) presented;

(legend continued on next page)

* $p<0.05$, *** $p<0.001$, **** $p<0.0001$ (one-way ANOVA followed by Tukey's multiple comparisons post-test). **(b)** Representative image of MDM pulsed with H1-VLPs at 45 min. Peripheral (towards the plasma membrane) re-distribution (recycling) of non-degraded HA can be seen. Green: fluorescently labeled HA, blue: nuclei stained with DAPI. Scale bar – 25 µm.

Supplemental references

1. Schneider, C. A., Rasband, W. S. & Eliceiri, K. W. NIH Image to ImageJ: 25 years of image analysis. *Nat. Methods* **9,** 671–675 (2012).
2. Pike, J. A., Styles, I. B., Rappoport, J. Z. & Heath, J. K. Quantifying receptor trafficking and colocalization with confocal microscopy. *Methods* **115,** 42–54 (2017).
3. Luisier, F. High-quality denoising of multidimensional fluorescence microscopy images (2D+t, 3D or color). *Biomedical Imaging Group (BIG), EPFL, Switzerland* (2014). Available at: http://bigwww.epfl.ch/algorithms/denoise/. (Accessed: 28th June 2018)
4. Castle, M. & Keller, J. Rolling Ball Background Subtraction. *Mental Health Research Institute, University of Michigan* (2015). Available at: https://imagej.net/Rolling_Ball_Background_Subtraction. (Accessed: 28th June 2018)
5. Collins, T. Colocalization Threshold. (2015). Available at: https://imagej.net/Colocalization_Threshold. (Accessed: 28th June 2018)
6. Costes, S. V *et al.* Automatic and quantitative measurement of protein-protein colocalization in live cells. *Biophys. J.* **86,** 3993–4003 (2004).
7. Dunn, K. W., Kamocka, M. M. & McDonald, J. H. A practical guide to evaluating colocalization in biological microscopy. *AJP Cell Physiol.* **300,** C723–C742 (2011).
8. Bolte, S. & Cordelières, F. P. A guided tour into subcellular colocalization analysis in light microscopy. *Journal of Microscopy* **224,** 213–232 (2006).
9. Macia, E. *et al.* Dynasore, a Cell-Permeable Inhibitor of Dynamin. *Dev. Cell* **10,** 839–850 (2006).
10. Delvendahl, I., Vyleta, N. P., von Gersdorff, H. & Hallermann, S. Fast, Temperature-Sensitive and Clathrin-Independent Endocytosis at Central Synapses. *Neuron* **90,** 492–498

(2016).

11. Preta, G., Cronin, J. G. & Sheldon, I. M. Dynasore - Not just a dynamin inhibitor. *Cell Communication and Signaling* **13,** 24 (2015).

12. Wang, L. H., Rothberg, K. G. & Anderson, R. G. W. Mis-assembly of clathrin lattices on endosomes reveals a regulatory switch for coated pit formation. *J. Cell Biol.* **123,** 1107–1117 (1993).

13. Daniel, J. A. *et al.* Phenothiazine-Derived Antipsychotic Drugs Inhibit Dynamin and Clathrin-Mediated Endocytosis. *Traffic* **16,** 635–654 (2015).

14. Heuser, J. E. & Anderson, R. G. W. Hypertonic media inhibit receptor-mediated endocytosis by blocking clathrin-coated pit formation. *J. Cell Biol.* **108,** 389–400 (1989).

15. Von Kleist, L. *et al.* Role of the clathrin terminal domain in regulating coated pit dynamics revealed by small molecule inhibition. *Cell* **146,** 471–484 (2011).

16. Willox, A. K., Sahraoui, Y. M. E. & Royle, S. J. Non-specificity of Pitstop 2 in clathrin-mediated endocytosis. *Biol. Open* **3,** 326–331 (2014).

17. Dutta, D., Williamson, C. D., Cole, N. B. & Donaldson, J. G. Pitstop 2 Is a Potent Inhibitor of Clathrin-Independent Endocytosis. *PLoS One* **7,** e45799 (2012).

18. Akiyama, T. *et al.* Genistein, a specific inhibitor of tyrosine-specific protein kinases. *J. Biol. Chem.* **262,** 5592–5595 (1987).

19. Tiruppathi, C., Song, W., Bergenfeldt, M., Sass, P. & Malik, A. B. Gp60 activation mediates albumin transcytosis in endothelial cells by tyrosine kinase-dependent pathway. *J. Biol. Chem.* **272,** 25968–25975 (1997).

20. Orlandi, P. A. & Fishman, P. H. Filipin-dependent inhibition of cholera toxin: Evidence for toxin internalization and activation through caveolae-like domains. *J. Cell Biol.* **141,**

905–915 (1998).

21. Koivusalo, M. *et al.* Amiloride inhibits macropinocytosis by lowering submembranous pH and preventing Rac1 and Cdc42 signaling. *J. Cell Biol.* **188,** 547–563 (2010).

22. Elliott, J. A. & Winn, W. C. Treatment of alveolar macrophages with cytochalasin D inhibits uptake and subsequent growth of Legionella pneumophila. *Infect. Immun.* **51,** 31–36 (1986).

23. Kuhn, D. A. *et al.* Different endocytotic uptake mechanisms for nanoparticles in epithelial cells and macrophages. *Beilstein J. Nanotechnol.* **5,** 1625–1636 (2014).
